# Strategic advances in liposomes technology: translational paradigm in transdermal delivery for skin dermatosis

**DOI:** 10.1186/s12951-025-03660-z

**Published:** 2025-08-21

**Authors:** Anmol Choudhury, Apoorv Kirti, Sudakshya S. Lenka, Shaikh Sheeran Naser, Adrija Sinha, Shalini Kumari, Nagendra Kumar Kaushik, Aishee Ghosh, Suresh K. Verma

**Affiliations:** 1 KIIT School of Biotechnology, KIIT University, 751024 Bhubaneswar, Odisha India; 2 Markham College of Commerce, Vinoba Bhave University, Hazaribagh, 825001 Jharkhand India; 3 Plasma Bioscience Research Center, Department of Electrical and Biological Physics, Kwangwoon University, Seoul, 01897 South Korea; 4 Department of Physics and Astronomy, Uppsala University, Box 516, Uppsala, SE-751 20 Sweden

**Keywords:** Liposome, Encapsulation, Transdermal, Drug delivery, Skin diseases

## Abstract

Liposomes, spherical vesicles made of phospholipids and cholesterol, have captivated researchers for their encapsulation abilities, biocompatibility, and versatility. This review delves into the core aspects and benefits of liposomal technology for enhancing transdermal drug delivery in treating skin dermatosis. It offers an extensive overview of liposomes, emphasizing various preparation methods, classification, and encapsulation techniques. The encapsulation of therapeutic compounds by liposomes boosts their utility as a stable and efficient drug delivery vehicle. Transdermal delivery presents a non-invasive alternative to oral and parenteral routes, allowing for controlled and sustained release of drugs while bypassing hepatic first-pass metabolism and minimizing systemic side effects. However, the stratum corneum acts as a formidable barrier to drug permeation. Liposomes, owing to their lipid bilayer structure that mimics skin composition, enhance drug solubility and partitioning, facilitate deeper skin penetration, and improve therapeutic efficacy, making them ideal carriers for transdermal applications. Transdermal delivery, favoured for its active and passive approaches and advantages over topical delivery, has been extensively studied and employed for therapeutics. Liposomes as delivery vehicles have significantly improved drug delivery efficiency and stability in transdermal applications. This review comprehensively examines the utility and mechanistic applications of liposomes. It also addresses the limitations and challenges in liposomal formulation that must be overcome for successful clinical trials.

**Graphical abstract:**

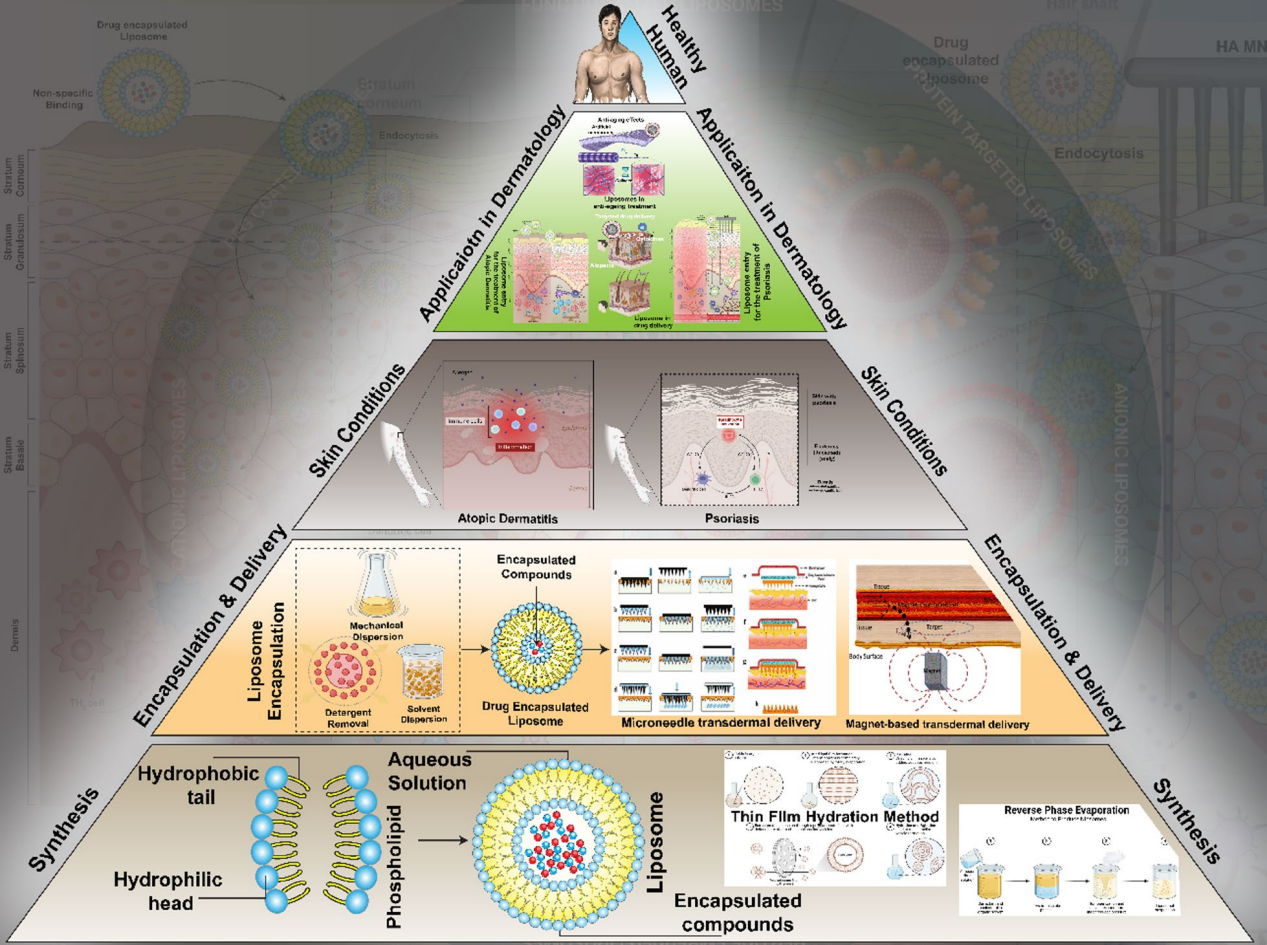

## Introduction

Nanotechnology has enhanced drug-delivery methods for the diagnosis and treatment of diseases. Owing to its multidimensional properties, researchers and industries have significantly emphasized liposome synthesis and design as drug delivery systems (DDSs). These DDSs may be customized to target drugs to the intended site of action precisely enhancing their half-life [1]. Alec D. Bangham, a prominent researcher at the Babraham Institute, University of Cambridge, discovered liposomes in the 1960 s, characterizing them as enlarged phospholipid structures [2]. The term liposome originates from the Greek words ‘lipos,’ signifying fat, and ‘soma,’ denoting body. It relates to phospholipid molecules, which function as the structural components of the body [3]. Liposomes have been extensively studied over the past fifty years due to their structural similarity to biological membranes and their ability to deliver various therapeutic substances [3]. A liposome is a self-assembled, bilayered lipid nanoparticle featuring an aqueous interior compartment, predominantly composed of biocompatible phospholipids and cholesterol. Comprehensive structural characteristics are detailed in Section"Liposome"[4].

Dermatosis is a skin condition that involves irritation and swelling, with symptoms including dry skin, rash, white patches, or blisters and flakes [5]. Skin dermatosis is initiated because of different reasons including viral, bacterial, fungal, or parasitic infections. Certain factors such as stress, allergies, and immune deficiency also play a major role in skin dermatosis [6]. The treatment of dermatosis has been explained through topical drug administration techniques over a century; however, transdermal delivery of drugs has been popularised because of the ability of the process to allow drugs to traverse across the stratum corneum, penetrating the dermis and epidermis, avoiding any drug accumulation.

Liposomes have been extensively explored as effective delivery vehicles for transdermal administration of drugs and organic compounds, owing to their structural similarity with biological membranes, excellent biocompatibility, and storage stability. They act as repositories for the gradual release of active substances and can penetrate the lipid bilayers of the epidermis and stratum corneum (SC) through individual phospholipids [7, 8]. Incorporating phospholipids into liposomal formulations enhances skin penetration and facilitates targeted drug delivery to specific sites of action. In dermatological applications, liposomes have demonstrated significant promise for both cutaneous and subdermal drug delivery, and their use in cosmeceuticals and cosmetics is steadily increasing. Their ability to be surface-modified allows for targeted delivery, while their biocompatibility, biodegradability, and minimal immunogenicity make them ideal carriers for both therapeutic and diagnostic agents [9, 10]. Liposomes also show a strong affinity for keratin in the stratum corneum, promoting deeper skin infiltration, higher absorption, and better drug retention, and they have been utilized as local anesthetics [11]. Additionally, nanotechnology-enabled vesicles such as transferosomes have proven effective in enhancing skin penetration of drug molecules. Liposomes have shown potential in improving the therapeutic outcomes of topically administered drugs for conditions such as psoriasis [12], while related systems like niosomes have demonstrated efficacy in treating psoriasis and hyperpigmentation [13, 14]. In acne treatment, lipid-based nanocarriers such as liposomes have been widely investigated for their ability to overcome the limitations of conventional formulations by enhancing permeation, targeting, solubility, and providing sustained release [15].

**Fig. 1:**
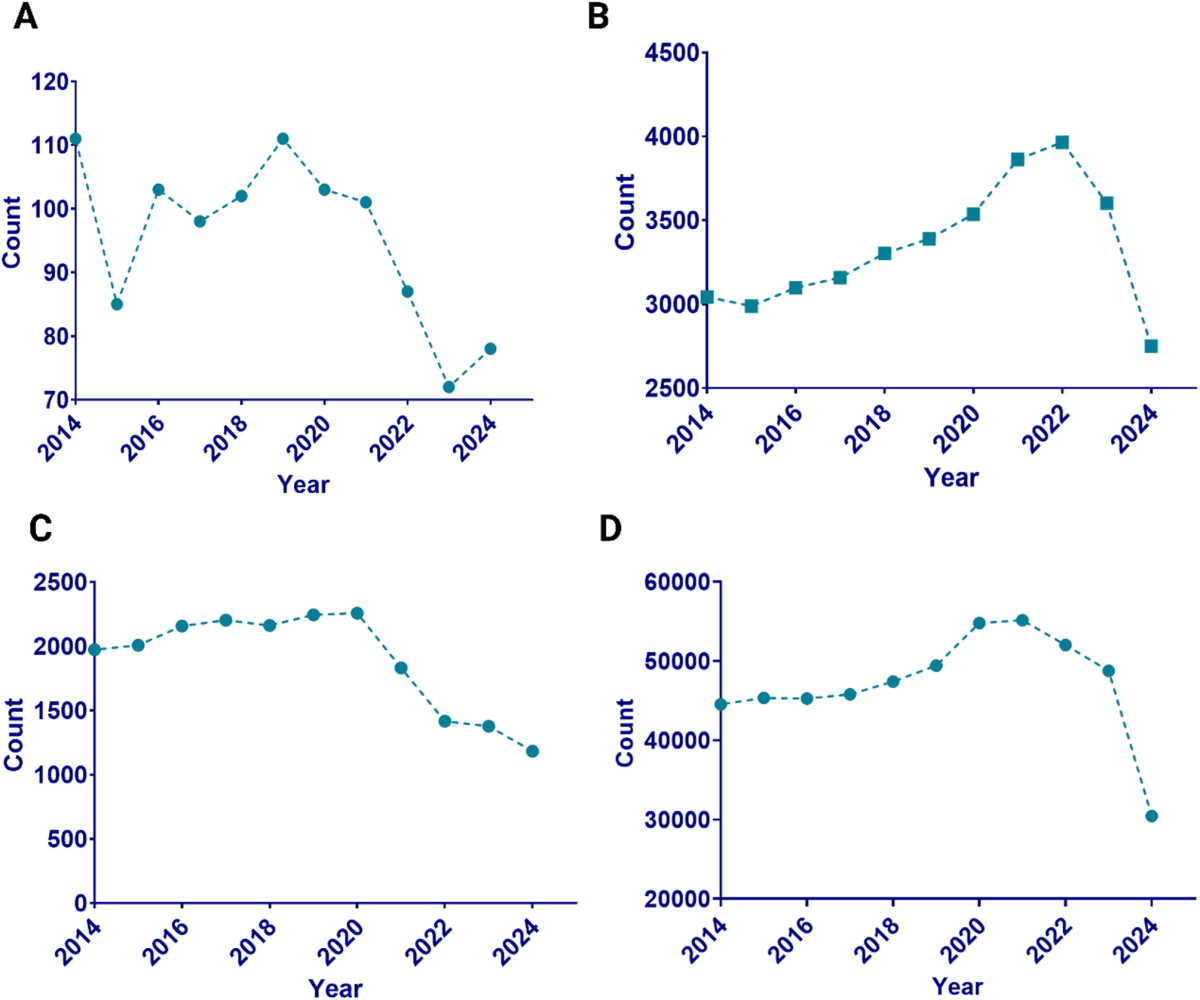
A detailed representation of the literature and studies done in the last 10 years on the topic **(A)** Liposomes in transdermal drug delivery for skin dermatosis, **(B)** Liposome, **(C)** transdermal drug delivery, and **(D)** Dermatosis

The literature on liposomes as transdermal drug delivery methods for skin dermatosis has experienced a significant rise in research studies over the last decade, indicating a growing interest in using these vesicular-carrying agents. Although much research focuses on liposomes in general, their use in the treatment of skin disorders is still an area with gaps that require additional investigation as evidenced by the literature published in the past decade (Fig. 1**)**. Although current research highlights the bioavailability and therapeutic effectiveness of liposomes, comparative studies indicate that there is currently a limited amount of literature specifically addressing liposomes for transdermal delivery in skin dermatosis. This indicates that while considerable advancements have been achieved, there is still much opportunity for further investigation, namely in the enhancement of formulations and comprehension of their intricate process of action. Emphasis on specific research could result in enhanced therapy approaches for a range of skin diseases, therefore addressing both effectiveness and patient adherence to treatment plans. This review delves into the properties of liposomes and their applications in transdermal drug delivery in skin diseases specifically skin dermatosis.

## Liposome

**Fig. 2:**
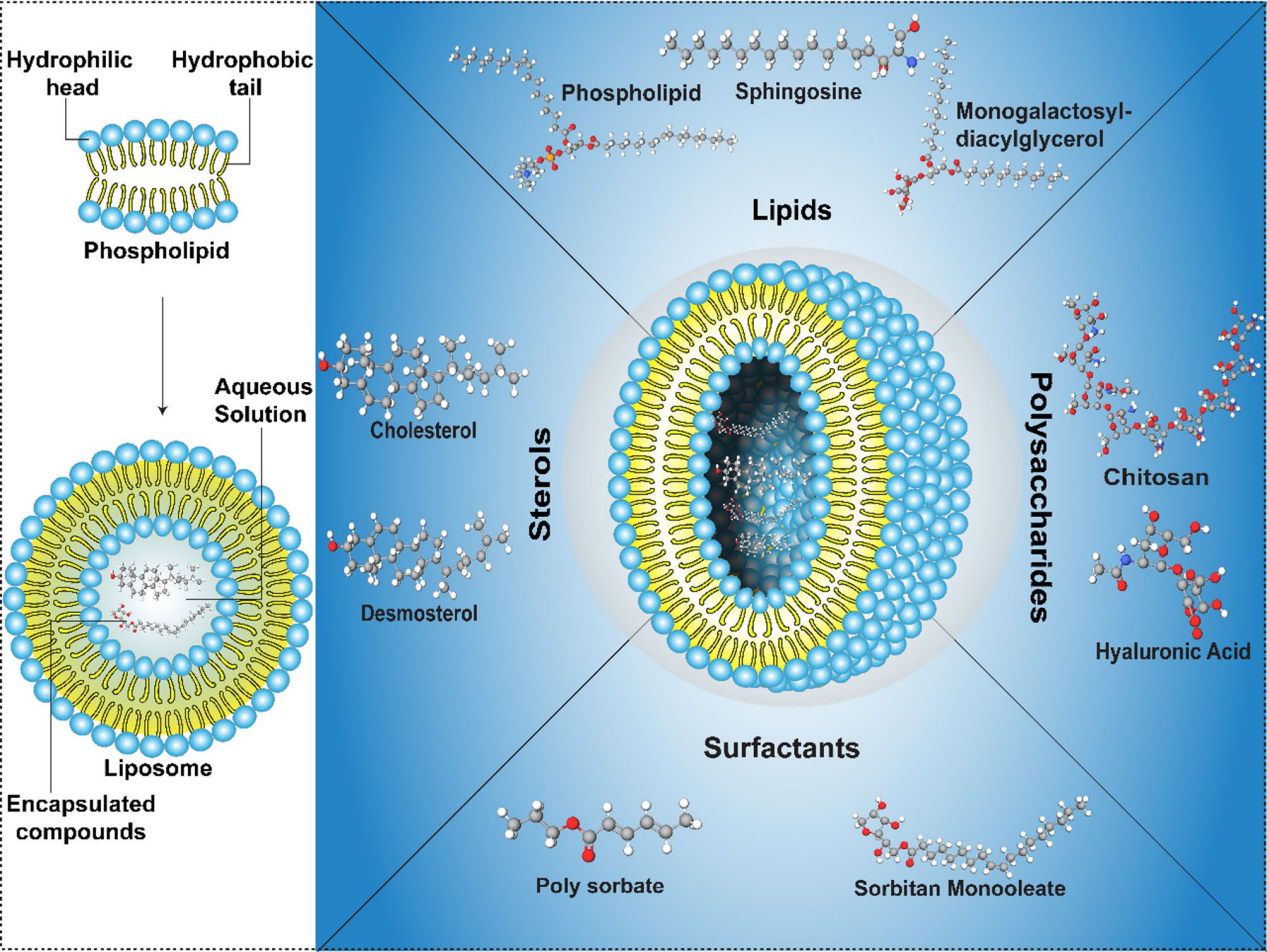
Schematic diagram showing the structure of the liposome and the various compounds encapsulated in the liposome

Liposomes are spherical vesicles formed by the fusion of different phospholipids. These are amphiphilic lipid nanoparticles, allowing the inclusion of hydrophilic and hydrophobic substances [16]. The primary components of a liposome are cholesterol and a non-toxic phospholipid. It has been reported to have a size range of 50–400 nm. The liposomes with a diameter of 150 nm ~ 200 nm have been demonstrated to be more spherically stable compared to those with sizes of less than 70 nm [4]. Studies are being conducted to develop techniques for optimizing the properties of liposomes for different applications [17]. The physicochemical properties of liposomes may be altered by varying the cholesterol and lipid concentration, method of synthesis, surface charge, and liposome size [16, 18]. Liposome molecules are composed of a hydrophilic head with two polar hydrophobic chains forming a membrane-like structure. The hydrophilic head of the lipid molecule can be classified as anionic, cationic, and neutral based on their respective charges (Fig. 2**)** [19–21]. The positively charged cationic head of the liposome enhances its attraction to the negatively charged cell membrane, hence increasing the efficiency of cell inclusion. Anionic liposomes have superior solution stability, reduced aggregation in comparison to neutrally charged liposomes, and enhanced endocytosis compared to neutral and cationic liposomes [22, 23]. According to research, the hydrophobic tail can influence the volume of the hydrophobic cavity and the ratio of the hydrophobic portion to the hydrophobic part of the liposome, hence influencing the liposome’s structure [24, 25].

Lipid vesicles are categorized based on their lamellarity into unilamellar vesicles (UVs), oligolamellar vesicles (OLVs), multilamellar vesicles (MLVs), and multivesicular vesicles (MVVs). Furthermore, unilamellar vesicles (UVs) can be further subcategorized based on their size into small unilamellar vesicles (SUVs) with diameters less than 100 nm, large unilamellar vesicles (LUVs) ranging from 100 to 1000 nm, and giant unilamellar vesicles (GUVs) exceeding 1 μm in size [26]. SUVs are characterized by a mono-phospholipid bilayer, whereas LUVs demonstrate an onion-like structure. Typically SUVs range from 20 to 100 nm in size, whereas LUVs vary from 0.1 to 1 μm, enabling significant encapsulation of hydrophilic substances within the aqueous compartments of the liposome [27]. MLVs range from 0.1 to 20 μm comprising of number of successive phospholipid bilayers. Several liposomes are combined to generate MVLs, which have a multilamellar structure with concentric phospholipid spheres [28]. For hydrophobic compounds, encapsulation efficiency is governed by both the liposome size and the number of bilayers. Typically, larger unilamellar vesicles provide greater bilayer surface area, facilitating enhanced drug incorporation. In contrast, multilamellar vesicles, with multiple concentric bilayers, may exhibit reduced loading efficiency due to restricted accessibility and lower available surface area per unit volume. The phase transition temperature (T_M_) of different natural and synthesized phospholipids differs, which is an essential metric in determining efficiency, drug encapsulation, stability, storage, and in vivo stability [29].

The physicochemical and colloidal properties of liposomes including their size, composition, loading efficiency, and stability are responsible for the benefits and limitations of liposomes as carriers for drugs [3]. The cell membrane interaction is also important in determining the benefits and limits of liposomes [30]. Liposomes and cells interact in four ways: phagocytosis, selective (receptor-mediated) or nonspecific endocytosis, absorption into the cell membrane, and local fusion (adhesion). Adsorption and endocytosis are the most predominant interactions among all. With endocytosis, liposomes and their contents are indirectly placed in the cytoplasm. Adsorption occurs when attractive forces prevail over repulsive forces, which is mostly determined by liposome surface characteristics [31, 32]. Another responsible interaction involves lipid exchange, a long-range process facilitating the transfer of bilayer components including cholesterols, lipids, and membrane-bound molecules between liposomes and cell membrane components [33, 34].

### Liposome preparation

The preparation of liposomes is followed by different approaches, including the use of different types of phospholipids. With each type of fabrication process and the phospholipid type, the final characteristics of liposomes can be altered. The first artificial liposomes were created by J. Y. Johnson in the 40’s and further patented the process for its application in the pharmaceutical sector (I. G. Farbenindustrie Aktiengesellschaft). When different types of fats or fatty oils were combined with varying water solutions, the resulting structures known as “depots” were created [35]. The liposomes can be prepared by employing various methods, some of which are mentioned below and in Table 1.

#### Thin film hydration method

The Bangham method, a commonly used approach in liposome synthesis, involves depositing lipid thin films on the inner surface of a rotary evaporator flask. During this procedure, lipids and hydrophobic compounds are dissolved in an appropriate organic solvent within a round bottom flask. Heterogenous liposomes are obtained on addition and mixing of the dispersion medium, above at the T_M_ [29, 32]. The substance that needs to be encapsulated is either introduced into the lipid formation or mixed with the buffer solution. The thin film hydration process is very reproducible, even with small amounts of chemicals, however, it has a low encapsulation efficiency.

#### Reverse-phase evaporation method

This is an alternative approach for the hydration method utilizing thin films, instead of employing a water-oil emulsion formation process. It encompasses the hydration of phospholipids dissolved within an organic phase, followed by the addition of water under intensive agitation [36]. Typically, a decreased concentration of phospholipids in the aqueous suspension results in higher production of LUVs relative to MLVs [37]. However, this method may induce denaturation in the therapeutic peptides due to exposure to organic solvents and sonication parameters, and is suitable only for molecules with high molecular weight. Extrusion can be employed to diminish both the average size as well as the polydispersity of preformed vesicles.

#### Solvent injection method

In 1973, Batzri and Kornfirst described the ethanol injection technique [38]. The general classification of solvent injection methods is based on the organic solvents employed. The phospholipids and their hydrophobic active agents are disseminated in an organic solvent. This mixture is then combined with an aqueous buffer, leading to the spontaneous formation of SUVs [39]. The addition of ethanol below its critical concentration to water induces the self-assembly of phospholipids, facilitating the formation of SUVs. Injecting ether leads to the formation of SUVs due to the evaporation of ether [40].

#### Detergent removal method

The method for liposome preparation was initially proposed for reconstituting the integral membrane proteins into phospholipid bilayers [41]. Here, surfactants with high micelle concentration (CMC) and lipids are dissolved in an organic solvent [42, 43]. Upon vigorous mixing, the detergents associate with the phospholipid forming the micelle structures. The surfactants are removed thereafter by dialysis, chromatography, size exclusion, dilution, or absorption onto hydrophobic beads giving richer lipids forming unilamellar vesicles [44].

#### Heating method

The heating method, developed by Mortazavi and Mozafari, is an organic solvent-free procedure [45]. Here, the lipids undergo hydration by an aqueous solution and are subjected to heating above the Tm of phospholipids in the solution in the presence of 3–5% hydrating agents like glycerine or propylene glycol [46]. The hydrating agent serves a dual purpose, acting both as a stabilizer and an ionizing additive, inhibiting the aggregation or settling of nanoparticles [46]. The Mozafari technique is an improved heating method for enhancing the stability of liposomes, in which the lipid components are hydrated in the aqueous medium before being heated in the absence of organic solvents [47].

#### pH jumping method

This is another solvent-free method that was first reported by Hauser and colleagues where SUVs were formed because of pH jump to MLVs [40, 48]. It is also known as the curvature-tuned method where phenomena such as the curvature theory of lipid bilayers and spontaneous vesiculation aid liposome formation. This method is comparatively faster with the production of stable liposomes [49]. In this curvature method, temperature, the duration of pH jump, and lipid composition are critical parameters that influence the polydispersity of the liposome and its size and shape.

**Table 1:** Lists of various methods for liposomes formation and their encapsulation efficacy

Method of liposome preparation	Description of method	Vesicles produced	Size of vesicles	Encapsulation efficiency	Reference
Bangham Method	The original liposome preparation the method involved the hydration of lipid film in an aqueous solution to form multilamellar vesicles.	MLVs	100–1000 nm	Moderate	[29, 40]
Ethanol/Ether injection	The lipid solution, consisting of lipids dissolved in a mixture of ethanol and ether, was introduced into an aqueous solution while being vigorously stirred. The outcome of this approach led to the creation of a solitary bilayer liposome, without the need for sonication.	SUVs LUVs	70–200 nm	High	[50–52]
Reverse phase evaporation	A method where an organic solvent containing lipids is emulsified with an aqueous phase containing drugs and then evaporated to form liposomes.	MLVs LUVs	100–1000 nm	High	[39, 53]
Curvature Tuning Method	A solvent-free technique that uses both charged and zwitterionic lipids combined with lyso- palmitoylphosphatidylcholine to create monodisperse solutions of very stable tiny unilamellar Vesicles. It involves the control of liposome size and shape by adjusting lipid composition to influence the curvature of the lipid bilayer by quick pH shift and equilibration interval.	Monodisperse SUVs	20 nm to 200 nm	Moderate to high	[39, 54]
Heating Method	Here, Lipids are heated above their transition temperature with glycerol, and PEG allowing them to form a homogeneous mixture. Upon cooling, the mixtures self- assemble into liposomes.	SUVs LUVs	50 nm- 500 nm	Low	[55]
Detergent Depletion	Detergents are gradually removed from a mixed micelle solution by dialysis, leading to the spontaneous formation of liposomes.	SUVs and LUVs	> 500 nm	Low	[45, 56]
Microfluidic channel	Liposomes are formed in this method by controlled mixing of lipid and aqueous phases present at the centre of dual channels, offering precise control over size and composition.	Monodisperse SUVs, GUVs, LUVs, MLVs	100–300 nm	Low	[53]
Freeze Drying of double emulsions	Double emulsions, composed of water droplets encapsulated within oil droplets, are subjected to freeze-drying (Lyophilization) to remove water, resulting in the formation of solid lipid nanoparticles or liposomes. It is used to encapsulate hydrophilic drugs with controlled release.	Monodisperse SUVs	200 nm- 5 µm	High	[57, 58]
Superficial fluids (SCFs) method	Liposomes are produced using supercritical fluids, such as carbon dioxide, to dissolve lipids, resulting in controlled size liposomes without organic solvents.	Monodisperse LUVs	100–300 nm or Or 0.2-4 µm	High	[40, 59]
Modified electroformation method	The process of liposome synthesis involves applying an alternating electric field to a lipid bilayer, which can be dry or damp, on an electrode made of ITO-coated glass slides. The electric field induces the membrane curvature and vesicle formation, followed by hydration to form liposomes.	Polydisperse GUVs	50 nm to > 100 μm	High	[60–63]
Size reduction of MLVs and GUVs	In this method, MLVs are resized to produce smaller liposomes by sonication, freeze-thaw extrusion, homogenization, and other techniques to produce either LUVs or SUVs.	Monodisperse SUVs and LUVs	40 nm to 200 nm	High	[64–66]
Biomimetic reaction for vesicular self-assembly	To replicate biological processes, biomimetic principles are used to produce liposomes, frequently with the assistance of catalyst enzymes. With the help of this method, vesicle production in aqueous solutions may be precisely controlled.	Polydisperse GUVs	20 nm to 10 μm	Moderate to high	[67, 68]
Hydration of phospholipids deposited on nanostructured material	The technique is used to create supported lipid bilayers through the deposition of phospholipids onto nanostructured materials ( electrospun amphiphilic nanofibre ), forming lipid bilayers through hydration.	Monodisperse SUVs and LUVs	50 nm- 500 nm	High	[69]

### Types of liposomes

#### Conventional liposomes

**Fig. 3:**
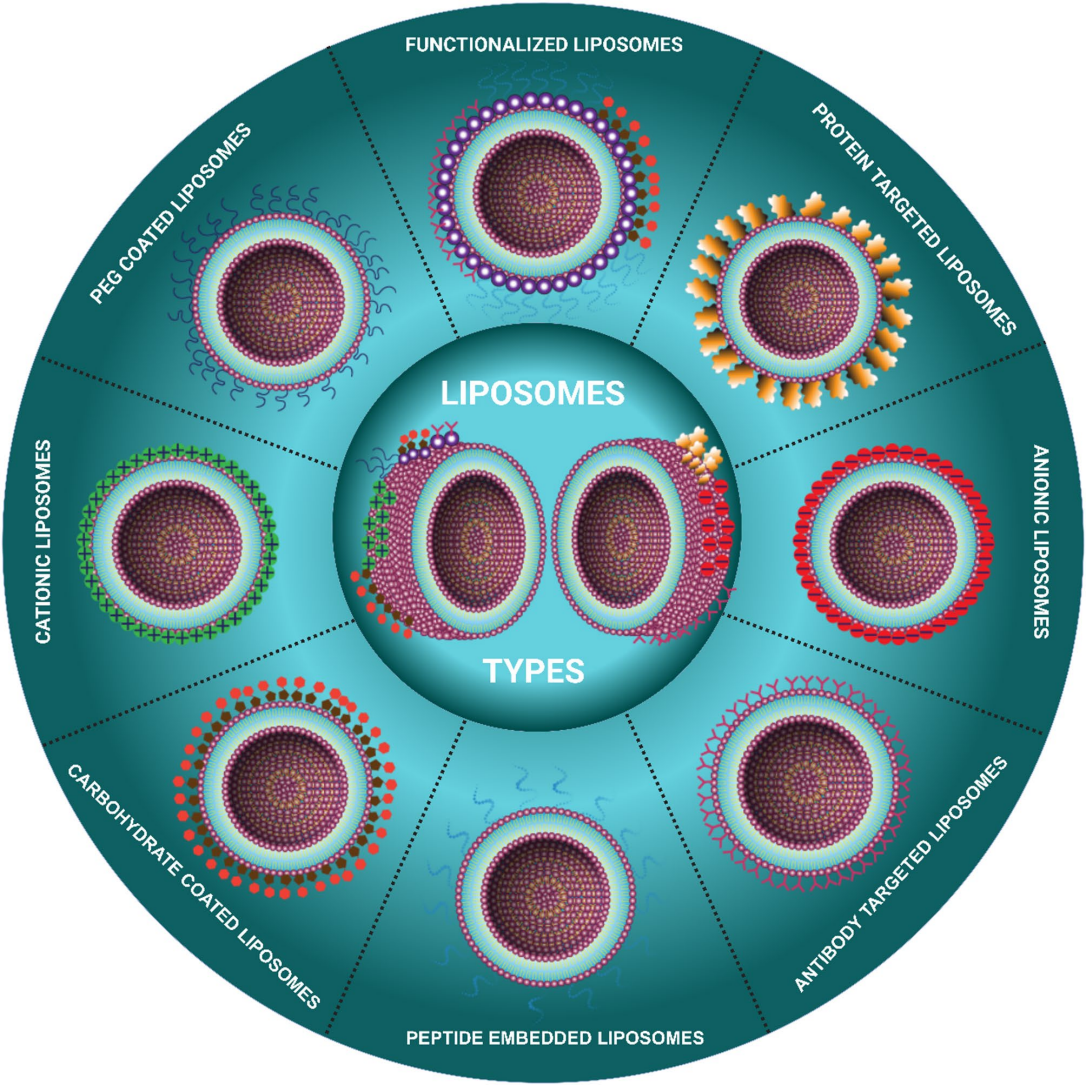
Schematic depiction of various types of liposomes offering significance advantages for targeted delivery, therapeutic efficiency, and biocompatibility

Conventional liposomes are spherical vesicles composed of a phospholipid bilayer. They are not coated with polyethylene glycol (PEG), which results in lower bioavailability compared to other well-established and emerging PEGylated liposomal formulations [70]. However, there are specific scenarios where non-PEGylated liposomes (NPLs) are preferred. Elan Pharmaceuticals has developed Myocet^®^, a non-PEGylated liposomal formulation of doxorubicin (NPLD), which has demonstrated reduced cardiotoxicity and enhanced bioavailability. Notably, NPLD has also mitigated the incidence of Hand-Foot Syndrome (HFS), a common side effect of conventional formulations. When combined with cyclophosphamide, NPLD is effectively used in the treatment of metastatic breast cancer [71]. Although PEG is not incorporated in conventional liposomes, the addition of cholesterol plays a critical role in maintaining liposomal membrane integrity. It modulates the conformational organization of lipid chains, thereby enhancing the stability of drug-loaded liposomes [72, 73]. Cholesterol influences key membrane characteristics such as strength, fluidity, stiffness, and permeability. It has been slos shown to impact the drug retention capability of liposomes through assistant in phospholipid packing (Fig. 3**)**.

#### Charged liposomes

Cationic liposomes are lipid-based carriers that have undergone extensive research for their varied applications, notably in drug delivery and gene therapy. They exhibit the potential to enhance the pharmacokinetics of drugs, such as tacrolimus, for the treatment of ocular diseases like dry eye [74]. Cationic liposomes have also been used as vectors for nucleic acids, including DNA and mRNA, in gene therapy applications [75]. Nevertheless, utilising these liposomes in cancer gene therapy faces limitations, both extracellular and intracellular, compromising their effectiveness [76, 77] Additionally, cationic liposomes have been found to enhance chemiluminescence reactions, and their positive charge plays a significant role in this process [78]. Overall, the properties of cationic liposomes, such as lipid composition, surface modification, and size, influence their performance and potential applications [79].

Anionic liposomes, which have a negatively charged surface, are being investigated extensively for drug delivery applications. They provide perks such as greater stability, improved drug protection, and extended drug release. They can be produced in various kinds of techniques, including organic solvents and dendrimer synthesis. These liposomes can be tailored for targeted administration to specific tissues or cells. Anionic liposomes, notably those made with glycerol or dendrimers, are widely recognized as safe and effective delivery vehicles because they have minimal toxicity and aid in the stability of nucleic acid drug therapies [80–82].

#### Stimuli-responsive liposomes

Stimuli-responsive liposomes are nanocarriers capable of releasing drugs in response to pH or temperature changes. One of the critical components in the synthesis of pH sensitive liposome is 1,2-dioleoyl-sn-glycero-3-phosphoenthanolamine (DOPE), however, DOPE being a non-bilayer lipid with a cone-shaped structure, does not form a bilayer alone. It can be stabilized in a bilayer by utilizing bilayer lipids such as phosphatidylserine (PS) or a weakly acidic amphiphile, cholesteryl hemisuccinate (CHEMS) [83]. Thermosensitive liposomes are synthesized by dipalmitoyl phosphocholine (DPPC), a phospholipid of 16 carbon atoms. Furthermore, its permeability and drug release capability can be enhanced by supplementing the liposome formation with distearoyl phosphocholine (DSPC) and hydrogenated soy phosphocholine (HSPC) [84]. Stimuli-responsive liposomes have low toxicity and excellent absorption in injured tissues, making them suitable for drug administration [82]. Liposomes that exhibit responsiveness to stimuli have a wide range of cargo release mechanisms. When triggered, a delicate characteristic in the liposomal bilayer causes the liposome to become unstable [85]. Parameters such as pH, temperature, light, ions, enzymes, and magnetic fields have the potential to induce the release of the payload [86]. The acidic environment of tumours can induce the release of the medication from pH-responsive liposomes. Ultrasound, as an external stimulus, can release payload [87, 88]. Ultrasound-responsive hydrogels, including colloidal structures such as liposomes, potentially modulate the spatial and temporal release of drugs, rendering them valuable in the fields of cancer treatment and tissue engineering [76, 86]. Stimuli-responsive liposomes facilitate targeted drug delivery to specific regions. The formation of stimuli-responsive liposomes depends on the deliberate choice and alteration of lipid components and supplementary elements that confer reactivity to specific stimuli.

### Thermosensitive system

Phospholipids constitute the structural foundation of stimuli-responsive liposomes. Natural or synthesised phospholipids, including 1,2-dipalmitoyl-sn-glycero-3-phosphocholine (DPPC) and 1,2-distearoyl-sn-glycero-3-phosphocholine (DSPC), are commonly utilised owing to their well-defined thermotropic phase transition properties. Thermosensitive liposomes typically consist of DPPC, which experiences a phase change at approximately 41 °C, facilitating the release of encapsulated medicines upon localised heating. The inclusion of monostearoyl phosphatidylcholine (MSPC) further improves thermosensitivity and leakage at the phase transition temperature [89].

### pH sensitivity

To confer pH sensitivity, lipids with ionizable group, such as 1,2-dioleoyl-sn-glycero-3-phosphoethanolamine (DOPE), are incorporated due to their fusogenic properties under acidic conditions. DOPE is often conjugated with cholesteryl hemisuccinate (CHEMS), forming a stable bilayer at physiological pH that becomes destabilized in acidic environments, such as endosomes or the tumor microenvironment, thereby promoting membrane fusion and facilitating drug release [83].

### Redox-responsive systems

Disulfide-containing lipids or polymer-conjugated lipids are utilized in redox-responsive liposomal systems. Disulfide bonds remain stable in the extracellular environment but are cleaved in the reductive intracellular milieu, characterized by elevated glutathione levels, leading to liposomal destabilization and subsequent drug release. Examples are distearoylphosphatidyl-ethanolamine (DSPE)-PEG conjugates altered with disulphide linkers (e.g., DSPE-SS-PEG), which provide responsiveness in tumour cells exhibiting heightened redox potential [90].

### Enzyme-responsive liposomes

Enzyme-responsive liposomes are constructed utilising substrates for specific enzymes overexpressed in diseased tissues. For example, matrix metalloproteinase (MMP)-sensitive peptides or phospholipids containing cleavable ester or amide linkages may be included in the liposomal bilayer. Enzymatic cleavage compromises membrane integrity, facilitating cargo release [91].

### Polymeric or inorganic additives

In certain instances, polymeric or inorganic additives are integrated to attain responsiveness. For instance, poly(N-isopropylacrylamide) (pNIPAM) can be grafted onto liposomal surfaces to achieve thermo-responsiveness, whereas gold nanoparticles can be included within or affixed to liposomes for photo-triggered release through plasmonic heating [92]. The underlying mechanism of responsiveness generally entails structural destabilisation of the lipid bilayer upon exposure to a stimulus, which increases membrane permeability or causes vesicle disintegration, enabling the regulated and site-specific release of encapsulated substances. These solutions collectively provide a method to enhance treatment efficacy while reducing systemic toxicity.

However, the development and application of stimuli-responsive liposomes in biological systems remain challenging [93, 94]. Key difficulties include optimizing liposomal formulation for stability and reproducibility, ensuring efficient stimulus-triggered drug release, and maintaining safety and biocompatibility [95]. Despite these obstacles, stimuli-responsive liposomes have intriguing biological uses [96]. They might revolutionise medication delivery, enable personalised therapy, and improve therapeutic efficacy and specificity.

#### Stealth liposomes

Stealth liposomes represent an advanced subclass of liposomal drug delivery systems engineered to evade detection and clearance by the mononuclear phagocyte system (MPS), thereby prolonging their systemic circulation. This immune evasion is primarily achieved through PEGylation, the covalent attachment of polyethylene glycol (PEG) chains to the liposomal surface. PEG forms a hydrophilic steric shield that minimizes opsonization and phagocytic uptake, significantly extending the circulation half-life of the encapsulated therapeutics [97, 98]. Some of the significant examples of stealth liposomes are Doxil/Caelyx (Liposomal doxorubicin) and SPI-077 (Liposomal cisplatin) [99, 100].

In contrast to conventional liposomes, which are rapidly eliminated from the bloodstream, stealth liposomes are capable of passive accumulation in tumor tissues via the enhanced permeability and retention (EPR) effect. This phenomenon arises from the characteristic leaky vasculature and impaired lymphatic drainage present in many solid tumors [101, 102]. Upon reaching the tumor site, stealth liposomes primarily remain within the extracellular matrix rather than entering tumor cells directly. Therefore, for drugs like doxorubicin or cisplatin to achieve therapeutic efficacy, they must first be released from the liposomal carrier into the tumor microenvironment and subsequently diffuse across the cellular membrane into the intracellular space [103]. Stealth liposomes have demonstrated notable versatility in encapsulating both hydrophilic and lipophilic drugs, protecting them from enzymatic degradation and improving pharmacokinetic metrics such as area under the curve (AUC) and maximum plasma concentration (Cmax) [104–106]. A well-established clinical example is PEGylated liposomal doxorubicin (PLD; Doxil^®^/Caelyx^®^), which is approved for the treatment of Kaposi’s sarcoma and recurrent ovarian cancer and is under clinical investigation for other malignancies, including breast cancer and glioblastomas [107].

Recent advances have focused on overcoming the limitations of PEGylation, often referred to as the “PEG dilemma”, where the PEG layer extending circulation time can hinder cellular uptake at the target site. To address this, researchers have developed stimuli-responsive PEG-shedding systems that react to tumor-specific cues such as acidic pH or elevated reductive potential, facilitating PEG detachment and enhancing cellular internalization [108, 109]. Taken together, these developments solidify stealth liposomes as a highly effective passive targeting platform. Their ability to accumulate at pathological sites through systemic distribution and physicochemical properties, without relying on specific ligand-receptor interactions, distinguishes them from actively targeted nanocarriers.

#### Active targeting liposomes

Active targeting liposomes are advanced nanocarrier systems designed to achieve site-specific drug delivery by leveraging molecular recognition mechanisms. Unlike stealth liposomes, which rely on the enhanced permeability and retention (EPR) effect for passive accumulation in diseased tissues, active targeting liposomes are functionalized with ligands that bind selectively to overexpressed receptors on target cells. This facilitates receptor-mediated endocytosis and enhances cellular internalization of the therapeutic payload [110, 111]. Various modifications can be employed for actively charging the liposomes, including immunoglobulin, carbohydrate, peptide, aptamer, protein, PEG, and small molecules such as folic acid and transferrin [112].

Targeting ligands may include monoclonal antibodies (e.g., trastuzumab targeting HER2), peptides (e.g., RGD motifs for integrins), aptamers, or small molecules such as folic acid [113–116]. Following receptor engagement, the liposome is internalized by the target cell, enabling efficient intracellular drug release. This ligand-directed approach reduces systemic toxicity, enhances drug bioavailability at the site of interest, and improves therapeutic outcomes, particularly in tumours with heterogeneous receptor expression profiles [117].

Functionalization techniques are broadly categorized into two strategies: post-insertion, where ligands are introduced to preformed liposomes; and one-pot assembly, which involves incorporating ligand-conjugated lipids during liposome formation. Each method offers distinct advantages with respect to ligand density, orientation, and liposomal stability [118–120].

Recent developments include dual-targeted liposomes, which employ two different ligands to recognize multiple tumor-specific receptors, thereby enhancing targeting accuracy in complex tumor microenvironments. Moreover, theranostic liposomes, which co-deliver drugs and imaging agents, have been developed to enable simultaneous therapy and real-time monitoring [121]. Active targeting liposomes have also advanced toward clinical applications. One prominent example is MM-302, a HER2-targeted liposomal doxorubicin formulation conjugated with trastuzumab fragments. In a Phase I clinical study, MM-302 demonstrated enhanced tumor-specific uptake and manageable safety in HER2-positive metastatic breast cancer patients [122]. Further development continued in the HERMIONE Phase II trial, although clinical success was constrained by tumor heterogeneity and variability in HER2 expression. Additionally, Du, J., Liu, X., Sun, J. et al. (2024) developed a novel trastuzumab-functionalized liposomal system for co-delivery of pyrotinib, demonstrating improved antitumor activity in HER2-overexpressing breast cancer models. Their preclinical evaluation revealed enhanced receptor-specific uptake and tumor regression, further supporting the viability of targeted liposomal delivery platforms in precision oncology [123].

Despite these promising advancements, significant challenges remain. These include immunogenicity associated with certain ligands, restricted tissue penetration due to the tumor’s dense extracellular matrix, and dynamic regulation of receptor expression that can hinder consistent targeting. Moreover, although many active targeting liposomes have shown efficacy in preclinical studies, the number of formulations reaching regulatory approval is still limited. Most approved liposomal therapeutics (e.g., Doxil^®^, Myocet^®^) utilize passive targeting mechanisms [124].

In conclusion, active targeting liposomes represent a powerful strategy for precision drug delivery, offering superior specificity and therapeutic efficacy. Continued innovation in ligand engineering, formulation optimization, and tumor microenvironment modulation is essential to enhance their translational success.

#### Ultra-flexible liposomes

Ultra-flexible liposomes such as transferosomes, ethosomes, and transethosomes possess high elasticity, enabling them to penetrate skin layers easily and deliver drugs to the desired site without any drug loss, enhancing bioavailability and therapeutic efficacy while decreasing their side effects [125]. Ethosomes are vesicular carriers constructed out of high concentrations of ethanol and phospholipids, designed specifically to improve drug delivery through the skin. They have presented significant potential in treating several disorders of the skin including, melanoma, acne, hair loss, and skin whitening [126–128]. These nanocarriers encapsulate a wide range of beneficial pharmaceutical components, making them ideal for administration through the skin [129]. Studies have demonstrated that ethosomal gels had higher transdermal flow and skin deposition than normal gels, proving their usefulness in delivering medicinal medications such as Spironolactone for acne treatment [130]. It has been studied that surfactants with suitable and correct amounts of lipid can destabilize liposome’s membrane which leads to vesicle membrane deformability. This characteristic of the edge activator (surfactant) was used in synthesizing transferosomes making it ultra-flexible and deformable [131]. The integration of Ethosomes and transferosomes results in transethosomes. The essential components of transethosomes are phospholipids, ethanol, and edge activator. Vesicular size, entrapment efficacy, and zeta potential are greatly dependent on the concentration and type of phospholipids [132]. Major advantages of transethosomes are better-bypassing first-pass metabolism, non-invasive drug administration technique making it more patient-friendly, and increased skin permeability, allowing deeper drug penetration. Compared to other ultra-flexible liposomes it is more biocompatible, stable, and biodegradable [133]. Challenges in this approach include achieving cell-specific and controllable delivery [22, 134].

## Encapsulation

Liposomes are one of the most suitable carriers with minimal toxicity and high accuracy of drug delivery in vitro. They act according to the pH and temperature of the environment and deliver its content. In context to subcutaneous and transdermal delivery, various compounds are being delivered currently. Encapsulation is a process through which a specific drug is enclosed within a vehicle to demonstrate in vivo, biodistribution, stability and pharmacokinetics. Attaining a high encapsulation efficiency is essential for a successful drug delivery. Drug encapsulation is preferred as it has the potential to circumvent some in vivo limitations of therapeutic compounds such as rapid clearance, poor biodistribution, low plasma solubility and poor pharmacokinetics. Despite there being various techniques to encapsulate the drugs and increase encapsulation efficiency, the freeze-thaw cycling and reverse phase evaporation techniques are used extensively [135]. These methods can seamlessly encapsulate hydrophilic and hydrophobic compounds into small liposomes (50–150 nm) [136]. However, while selecting a suitable protocol for encapsulation, various factors are checked, such as drug/lipid ratio, sterility, drug retention, encapsulation efficiency, and drug stability [137].

**Fig. 4:**
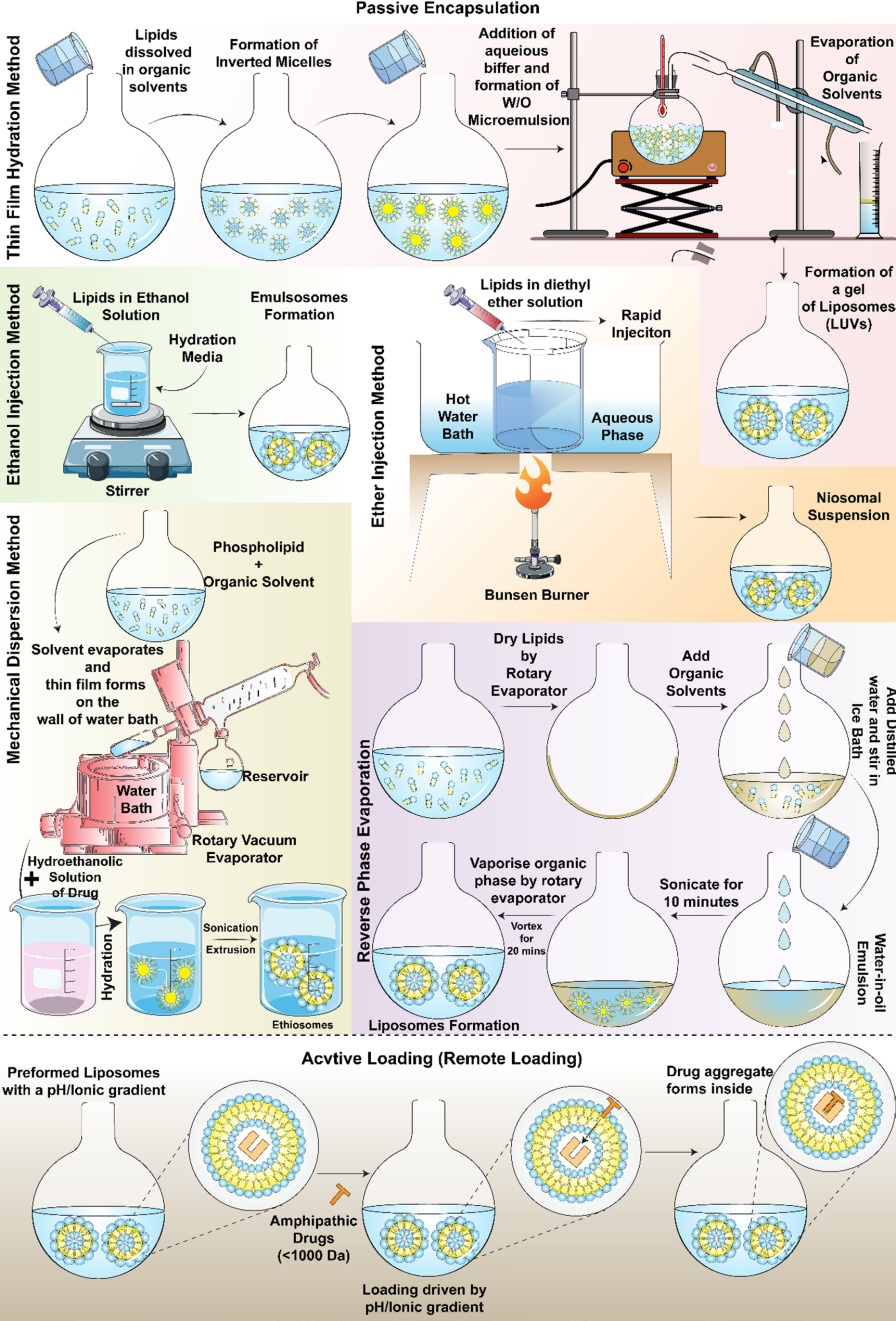
Schematic diagram illustrating major processes involved in the passive and active loading in liposomes

When liposomes are selected, the vesicle size is chosen based on the site of action as, while administering a compound into the lungs, it has been found that injection of MLVs (d = 3 μm) enhances the delivery [138]. Nevertheless, increased partitioning of the bone marrow is seen after administering SUVs [139]. Several studies have shown that due to the interaction with high-density lipoproteins, vesicles that are composed of no more than one phospholipid, become leaky in plasma or serum. Therefore, to overcome this, cholesterol is incorporated into the lipid bilayer which also provides additional improved stability to the liposome while circulating in the blood vessel [137, 140]. Techniques extensively utilized for encapsulation in liposomes are active loading and passive loading techniques. Passive loading techniques depend on the ability of the liposomes to encapsulate a certain amount of aqueous solute and solvent during the formation of the vesicle, whereas active loading techniques involve methods for encapsulating amphiphilic compounds as they can rapidly permeate through the lipid bilayer. The lipid composition of liposomes is altered to enhance encapsulation and decrease the rate of release [137]. Figure 4 shows the mechanism of some of the passive loading and active loading methods in liposomes.

### Passive loading in liposome

Passive loading techniques are usually employed to entrap the materials which act completely as aqueous markers as well as materials which are water-soluble bioactive agents. Types of vesicles used such as MLV, SUV or large unilamellar vesicles (LUV) and their encapsulation efficiency varies greatly from 1% for SUVs to 88% for MLVs. This is because SUVs can entrap at lower lipid volume i.e., 0.2–0.8 µl/µmol than compared LUVs and MLVs which show higher lipid entrapment volumes i.e., 1–30 µl/µmol. Due to this varying encapsulation efficiency, not all markers that researchers want to encapsulate act as an ideal aqueous marker [137].

Passive loading techniques encompass three distinct methods commonly used for encapsulation such as solvent dispersion method, mechanical dispersion method and detergent removal method.

### Solvent dispersion method

Lipids are dispersed in a solvent and then they are gradually mixed into an aqueous phase. Three types of solvent dispersion methods are currently being used, such as ethanol injection, ether injection, and reverse phase evaporation method.

#### Ethanol injection method

requires a lipids and ethanol solution to be introduced into an excess amount of buffer. This process immediately creates MLVs with varying sizes (30–110 nm). The liposomes formed have modest concentrations, and the elimination of ethanol can be difficult due to their formation of an azeotrope with water [38].

#### Ether injection (Solvent vaporization)

entails introducing diethyl ether and lipids solution or ether-methanol into an aqueous solution containing the target molecule for encapsulation. This is done at a temperature range of 55℃ to 65℃ or under reduced pressure conditions. Under vacuum conditions, ethanol is gradually eliminated leading to the encapsulation of the compound in the liposome. However, the size of the liposomes is not controlled, and the liposomes formed are of varying sizes (70–200 nm) [141].

#### Reverse phase evaporation

is a technique that relies on the formation of inverted micelles. During this procedure, an organic phase solution containing amphiphilic molecules is mixed with a solution containing the compounds to be encapsulated and then subjected to sonication. A viscous or gel-like structure forms because of the gradual release of an organic solvent, and this gel collapses at a critical stage in the process, disrupting certain inverted micelles. This process helps in generating liposomes utilizing the surplus phospholipids from the disrupted micelle to build a bilayer structure within the remaining micelle [18]. However, sonication of the solution might result in protein and DNA denaturation.

This approach produces MLVs with equilibrium transmembrane solute distribution that retain chemicals for longer and trap 6–38%. Thus, solvent dispersion has been widely employed to encapsulate drugs in LUVs due to its high trapping efficiency of 30–45% and ability to accept a larger compound volume with a high drug/lipid ratio [40]. Encapsulating RNA, proteins, and DNA is possible using this approach.

### Detergent removal method

The detergent removal method basically involves detergent extraction from the solution leading to the formation of liposome. This is achieved by detergent removal of mixed micelles, dialysis, and gel permeation chromatography.

#### Dialysis

is the process through which the detergent is removed by a device known as LipoPrep (Diachema AG, Switzerland) [142]. Detergents get detached resulting in the combination of micelles with the phospholipid and formation of LUVs. Detergents are employed to solubilize lipids at their critical micelle concentration (CMC) [18].

#### Detergent removal of mixed micelle

is the adsorption technique where detergents (alkyl glycosidase, Triton X-100, cholate) are kept in a shaking condition with beads of adsorbers functionalized on micelles. Adsorbers are composed of organic polystyrene materials, such as XAD-2 and Bio-beads SM2. This approach can facilitate removal of detergents with a comparatively lower CMC [18].

#### Gel-permeation chromatography

exploits the difference in size between detergents and liposomes. Beads of Sephacryl S200-S1000, Sephadex G-1-00, Sepharose 2B-6B and Sephadex G-50 can be used for size exclusion chromatography. Liposomes are bigger in size; allowing them to pass through the inter-bead spaces [143]. LUVs display variability in drug/lipid ratio and can encapsulate higher volumes (1.5–30 µl/µmol), while their size ranging between 0.1 μm and 10 μm. This method is used to encapsulate SUVs and LUVs, allowing us to adjust liposome size by altering the detergent/lipid ratio and enhancing encapsulation efficiency.

### Mechanical dispersion method

The mechanical dispersion method is the most used method for encapsulating drugs in the liposomes. It involves physical methods to disrupt lipid bilayers and then encapsulation is carried out. This method involves Freeze-thawed liposomes, sonication, and French pressure cell: extrusion.

#### Freeze-thawed liposomes

involve quick freezing and gradual thawing of SUVs followed by sonication of the aggregates which disperses them resulting in the formation of LUVs [144]. The inhibition of synthesis can be achieved by increasing the concentration of phospholipid and the ionic strength of the medium. This approach ensures an encapsulation efficiency ranging from 20% − 30% [145]. Though this technique is designed for LUVs, MLVs may encapsulate other chemicals without harming DNA, RNA, or proteins if executed properly with up to 88% entrapping efficiency.

#### Sonication

results in the conversion of MLVs into SUVs. It is the most used method in which the liposome dispersion is sonicated which leads to the encapsulation of the compounds. Two predominant sonication techniques are being used extensively: bath sonication and probe sonication. In the process of bath sonication, the dispersion is contained within a cylindrical container placed inside a bath sonicator, whereas the probe sonication method utilises the tip of the probe being directly immersed in the dispersion. Bath sonication is preferred as probe sonication might release heavy metals like titanium in the dispersion [18]. This method has a low encapsulation efficacy and high frequency can degrade the compounds that are to be encapsulated.

#### French pressure cell extrusionis

a process in which the MLVs are extruded through a small orifice such as 0.5 μm. The vesicles formed by the French press have better encapsulation efficiency as they seem to remember entrapped compounds for a longer period [146]. The unstable materials need to be handled carefully and the liposomes formed are larger than the ones created by the sonication method. This method requires high temperatures which is difficult to attain as well as only small working volumes can be used such as 50mL maximum.

### Active loading in liposomes

Unlike passive loading, active loading methods involve procedures in which drug/lipid ratios of the solution are greater than the calculated aqueous trapped volumes. Ion gradient drug loading allows amphiphilic cations like Adriamycin to be encapsulated. This mechanism resembles the way particular probes relocate across lipid bilayers in response to ΔΨ and ΔpH changes [146]. The pH-dependent drug uptake mechanism functions similarly to the ΔpH-dependent transmembrane redistribution of weak bases. Unprotonated ions permeate the lipid-bilayer and accumulate inside the vesicle until [AH^+^]_in_/[AH^+^]_out_ = [H^+^]_in_/[H^+^]_out_, where AH + is the compound’s protonate form. Encapsulating drugs across the ion gradient boosts trapping efficiency and reduces drug efflux from vesicles by 30-fold [147].

Nicols and Deamer were among the first researchers who demonstrated active loading of amphiphilic amine catecholamines in liposomes [148]. A pH gradient was created by using citrate buffer of 3 units 8.0 outside and 5.0 inside the liposome. To further explore this citrate method Bally et al., studied the effect of proton gradient by replacing the intraliposomal solution to HEPES buffer pH (7.0) from 300 mm citrate buffer (pH 4.0), keeping the internal pH undisturbed. It was observed that biogenic amines such as doxorubicin (anticancer drug) accumulated in the solution [149]. Moreover, it had been seen that a 100% encapsulation efficiency with a high stability was achieved when idarubicin, daunorubicin and doxorubicin were loaded in drug/lipid ratio of 0.3 w/w [150].

Gradient of pH can be generated by using ionophore, which is another versatile approach for encapsulation. Deamer et al. were the first to identify that SUVs containing potassium would generate pH gradient of 2 units if nigericin was added to it [151]. To facilitate the absorption of drug, the ionophore is added which couples the outward movement of metal ions and the inward movement of H^+^ ions. pH gradient is created which leads to the uptake of weak base molecules. Ionophores offers the opportunity of changing the internal ion which can facilitate different drug transport such as, magnesium or calcium ions. These ions can be used to achieve the required pH gradient as well as for the formation of metal-drug complexes [152]. This is how drug release kinetics can be altered depending on their solubility and the pH inside and outside the membrane.

Based on the available information, it is evident that liposomes can encapsulate a diverse range of components. The process of encapsulation and subsequent release is dependent upon the polarity of the compounds involved. It is important to note that not every drug or compound can be effectively encapsulated within liposomes, and each molecule will require a distinct method to ensure successful encapsulation.

### Stability

Liposomes can be subjected to degradation due to the formation of self-aggregates, flocculation, the occurrence of coalescence, and precipitation of aqueous liposomes during synthesis and storage [153]. Such instabilities may compromise the integrity of the vesicular structure, resulting in premature drug leakage and uncontrolled release kinetics. Several physicochemical parameters critically influence the membrane fluidity and, consequently, the physical stability of liposomes [154, 155]. These include temperature, pH, ionic strength, the composition of constituent phospholipids, and the incorporation of stabilizing agents within the lipid bilayer. Additionally, factors such as vesicle size, surface charge, and osmotic pressure contribute to membrane integrity and overall formulation robustness [156].

To assess liposomal stability, both long-term and accelerated studies are conducted, employing techniques such as Dynamic Light Scattering (DLS) and advanced microscopy, including cryogenic electron microscopy (cryo-EM), for the evaluation of particle size and distribution. Stability assessments following external stressors, such as centrifugation, heat exposure [157], or surfactant treatment [158], provide further insight into formulation resilience. Computational approaches, including molecular dynamics simulations, have also been utilized to explore the influence of lipid composition and membrane curvature on structural stability over extended periods [154, 156].

Beyond physical parameters, the chemical stability of liposomes is influenced by the susceptibility of lipid functional groups to oxidative and hydrolytic degradation. To mitigate oxidation, raw materials must be free of reactive impurities such as transition metal ions and peroxides [159]. Optimal storage conditions include oxygen-free environments, protection from light, and maintenance at reduced temperatures. Moreover, the incorporation of antioxidants such as butylated hydroxytoluene (BHT) and α-tocopherol has proven effective in preserving phospholipid integrity by inhibiting oxidative processes [154, 160, 161].

**Fig. 5:**
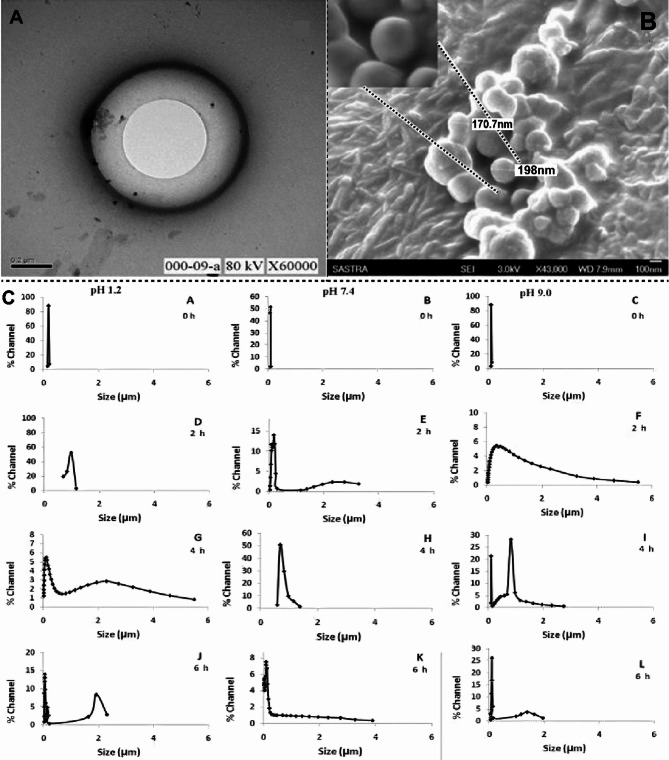
TEM and SEM micrographs of lyophilized liposomes: (**A**) TEM Image was taken at a magnification of 60,000, (**B**) SEM image at a magnification of 43,000 and the callout image represents the magnified image of the liposome. (**C**) Graphs represents the stability of liposomes in different pH. Adapted with permission from [167]

Post-processing techniques are employed to generate dry and stable liposomes which are highly stable, and the contents are more accessible than the aqueous liposomes. Techniques used include spray drying, freeze-drying, and spray freeze-drying. Figure 5 shows the electron microscopy images of liposomes and the stability of liposomes at different pH during their formation.

#### Freeze drying process

It is also referred to as lyophilization, which eliminates water from the liposome solution. It involved three primary steps: (1) Liposome freezing with a cryoprotectant, (2) Primary drying and sublimation, and (3) Secondary drying to remove the remnant moisture [162]. This process is mandatory as it prevents the encapsulated compounds from degradation, and it also ensures the stability of the liposomes [163]. Despite the added advantages of the freeze-drying method, there are a few disadvantages associated with it such as membrane integrity may be lost, encapsulated substances might leak out from the vesicle and vesicle size might decrease [163]. Research conducted by Franze et al. reported that during rapid freezing, small, iced crystals can form that can minimise disruption of the bilayer structure. However, large ice crystals are formed, if slow freezing is conducted, damaging the bilayer structure [164]. Despite this drawback, various situations might be created where slow freezing must be required, hence, to overcome this lyoprotectant should be added to the liposome mixture. Lyoprotectants prevent bilayer damage, leakage of encapsulated materials and vesicle aggregation. Proteins, carbohydrates, and alcohols are some of the compounds that act as lyoprotectants [153].

#### Spray drying process

Spray drying process is used to convert aqueous liposomes into dried and stable form and is preferred over freeze drying because it is comparatively affordable, and particles of defined sizes can be formed [165, 166]. Despite this, there are some limitations such as after drying a particle it starts to form aggregate increasing in size of liposomes which eventually leads to leakage of its contents. The formation of aggregates can be checked by utilizing oppositely charged biopolymers such as carbohydrates and proteins as protectants to improve mechanical stability and kinetics.

#### Spray-freeze drying process

This process is being widely used for the improvement of encapsulation efficiency and stability of liposomes. It amalgamates the benefits of freeze-drying and spray drying, resulting in highly stable products [164]. Three major steps are involved in the process: Atomization, freezing, and freeze drying. An atomizer is used, which converts the feed solution into spherical droplets followed by freezing and freeze-drying using a cryogenic agent. Sublimation of the ice is carried out at low pressure and temperature. The product’s physicochemical characteristics alter based on the equipment type being used [164].

### Targeted drug delivery (TDD)

Targeted drug delivery stands as a meticulous approach, directing medication accurately to afflicted cells or tissues, thereby amplifying their therapeutic impact while mitigating adverse effects on healthy cells or tissues [168, 169]. TDD improves drug concentration at the targeted location while minimizing side effects and strengthening treatment efficacy [170]. The swift progress in interdisciplinary technology and the creation of effective nanomedicines, possessing safety profiles and potent targeting abilities, promote their clinical use, contributing invaluable support to medical practitioners and patients [168]. Nanomaterial-based drug delivery systems (NBDDS) utilize nanoparticles for improving drug effectiveness and safety. This approach represents a significant breakthrough, enabling the scientific community with tools to tackle the shortcomings inherent in traditional delivery systems with conventional drug administration [171]. NBDDS utilize rational targeting pathways to enhance stability, bioavailability, solubility, and cell membrane transport, and minimize adverse reactions. TDD using liposomes involves encapsulating drugs within phospholipid bilayer vesicles, allowing for efficient delivery to specific sites in the body [172]. Liposomes can be synthesized through diverse methodologies like mechanical dispersion and solvent dispersion. Moreover, they can be tailor made to modify drug release rates and enhance encapsulation efficiency [173].

Factors influencing drug targeting involve the type of targeting, targeting moieties, targeting sites, drug carriers, and ligands. Passive targeted delivery involves the use of carrier systems that prolong residence time and increase bioavailability, leading to higher therapeutic efficacy. This approach does not involve the selective binding of drug molecules to receptors of the targeted sites. Active targeted delivery, on the other hand, utilizes drug molecules that selectively bind to receptors of the targeted sites, allowing for specific delivery to the desired tissue or organ. Active targeting can improve drug distribution and minimize unwanted side effects. It is mostly used to treat cancer, cardiovascular illnesses, neurological ailments, and respiratory diseases [174–176]. Using passive targeting to create nano-carrier structures containing chemotherapeutic drugs can improve Drug delivery and patient survival rates. Nanoparticles can reach the tumour microenvironment and deposit inside it through passive targeting, which utilizes the aberrant vasculature of cancerous cells [177, 178]. While TDD systems have demonstrated promising outcomes, scaling up, translating preclinical research into clinical success, and obtaining regulatory approval remain difficulties for commercial success.

To effectively treat skin cancer and various other dermatological conditions, TDD is an promising strategy. Nanotechnology has the potential to offer secure and customised delivery of drugs. Nanocarriers have been shown to enhance the delivery of dermatological medicines. The physicochemical characteristics of nanoparticles facilitate the delivery of drugs and enhance skin permeation [179]. Several specialized targeted nanocarriers, such as liposomes, dendrimers, inorganic nanoparticles and inorganic nanoparticles, have been developed for skin cancer treatment [170]. The use of FDA-approved excipients and innovative formulation platforms has been explored to optimize the properties and permeation behaviour of drugs in the skin [180]. TDD in dermatology aims to prolong, localize, and target drug interactions with the diseased skin, leading to improved treatment outcomes and quality of the patient’s life [181]. However, there are still challenges that need to be addressed, such as the optimization of properties, reliable characterization methods, safety evaluation, nanoparticles that might lead to unintended consequences like nano-pollution and cytotoxicity and regulatory aspects related to nanomedicines targeting skin cancer [176, 179].

## Liposome for dermatosis treatment

Dermatosis is a general term for all the skin defects, or any lesions formed on the skin and can be caused throughout the body and not specific to any single part [182]. Dermatotic conditions such as tumors, chronic wounds and psoriasis are triggered due to the perturbation of skin homeostasis because of both internal and external factors. Barrier dysfunction due to unavoidable injuries or allergic responses enhances the release of IL-1α, leading to the increased formation of cytokine proteins [183, 184]. This hypothesis suggests that inflammatory cells phenotype specific like the abnormal T cells are trapped within the skin barriers resulting in the release of signalling molecules from the epidermis. A cascade mechanism gets initiated leading to the downstream stimulation of chemokine and intracellular adhesion molecule (ICAM) formation following the generation of IL-lα and TNFα, respectively [185].

**Fig. 6:**
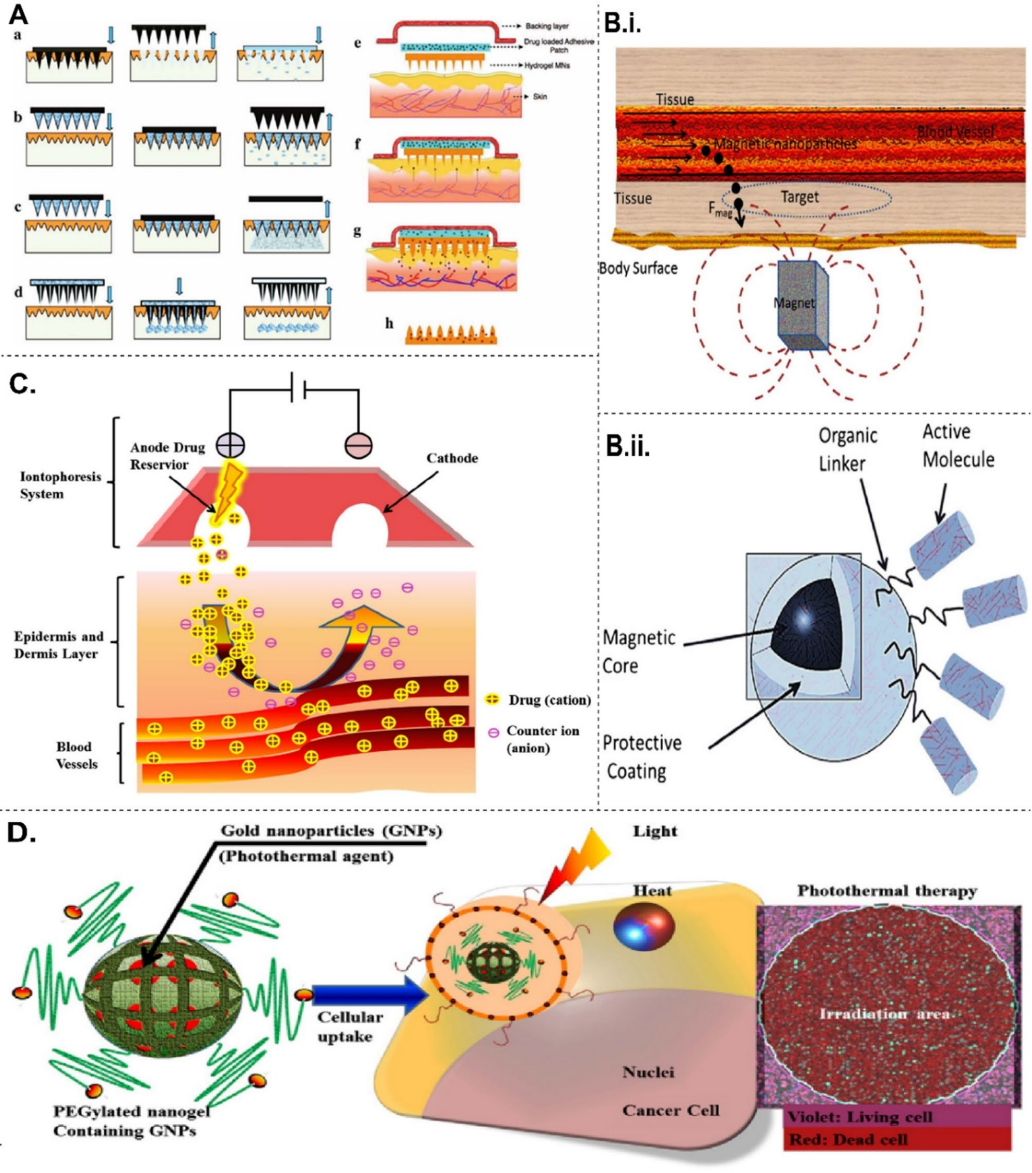
**A**. Diagram illustrating techniques for applying microneedles in the skin to improve delivery of drugs through the skin. **(A) **a-d shows conventional approaches to drug delivery using microneedles. **(A) **e-h introduces hydrogel-making polymeric microneedles (Adapted with permission from [191]). (**B).**i. Depicts drug delivery system utilizing magnetic nanoparticles. ii. A typical magnetic nanoparticle comprises a magnetic nucleus, a shielding layer, an organic connector, and an active compound. **C.** This image illustrates the iontophoresis method. **D.** Illustration shows the electroporation phenomenon. **E.** This illustrates Photothermal therapy. Cells internalize PEGylated nanogels incorporating gold nanoparticles. Both heat and light irradiation are lethal to cancer cells. (**B-E** Adapted with permission from [192])

The continuous distribution in the skin barrier due to genetic factors, injuries, infections, or immune dysregulation is termed inflammatory dermatoses [186–188]. The continuous disruption leads to kelpids and hypertrophic scars which can be viewed as an exuberant wound healing response, explained by a primary barrier abnormality [189]. Hardening refers to the process of repairing a barrier to compensate for any existing damages. This condition exhibits reduced levels of irritation; however, it is characterised by inflammation and epidermal hyperplasia. In summary, this outside-inside theory of skin dermatosis, which encompasses irritating chronic dermatitis, holds significant therapeutic implications [190]. Figure 6 represents various techniques being employed for the transdermal delivery of drugs.

### Application of liposomes in dermatology

Liposomal formulations have shown considerable promise in treating numerous dermatological problems, such as infections, inflammatory disorders, and neoplastic lesions, by boosting medication penetration, improving bioavailability, and minimising systemic side effects. Table 2 summarizes key studies done employing using various liposomal formulations.

**Table 2:** Various liposomal formulations for the treatment of different dermatological diseases

S.No.	Liposomal Formulation	Dosage	Disease Targeted	Observed Outcomes	Adverse Outcomes if any	Clinical or Preclinical Studies	References
**1.**	Azithromycin loaded liposomes	250 mg twice/day	Cutaneous Leishmaniasis	• Decrease in Lesion size observed. • Slight improvement: 25% lesion size decrease. • Mild improvement: 25–75% decrease in lesion size. • Moderate improvement: 50–75% lesion size decrease. • Significant improvement: 75% decrease in lesion size.	Mild Pruritus in 2 cases	Pilot Clinical Study: randomized, open label, parallel-group trial	[193]
**2.**	Fluconazole and Terbinafine loaded liposomes	Fluconazole nanoliposome: 0.5% Terbinafine nanoliposome: 1%	Dermatophytosis	• Faster lesion clearance. • Complete clinical recovery by day 40.	No adverse effect	Pre-clinical study conducted on Guinea Pigs	[194]
**3.**	Irutinib and Curcumin loaded liposomes	Irutinib: 10 mg/kg Curcumin: 20 mg/kg	Psoriasis	• Epidermal hyperplasia and ear thickness were reduced significantly. • Psoriasis Area and Severity Index (PASI) score were significantly decreased. • Inflammatory cytokines (TNFα, IL-17, and IL-32) decreased significantly	No reported adverse effect	Preclinical study on BALB/c mice	[195]
**4.**	Amphotericin B loaded Liposome	200 mg, 400 mg, and 800 mg	Mucocutaneous Candidiasis	• All patients exhibited clinical improvement within 2 weeks, in which 3 received 400 mg and 1 received 200 mg daily. • 3 patients maintained sustained response for 60 months. • 1 patient experienced relapse at 24th week and was withdrawn.	No serious adverse effect observed	Clinical trial phase 2	[196]
**5.**	Amphotericin B loaded liposomes	0.4% gel applied twice daily for 28 days	Cutaneous Leishmaniasis	• 19 of 22 patients (36 of 39 lesions) were completely cured within 42 days. • 95% of efficacy achieved.	Mild local burning sensation were reported in a small number of patients less than 30%	Clinical trial phase 2	[197]
**6.**	*Vaccinium vitis-idaea* extract loaded liposome	N/A	Atopic Dermatitis	• AD = associated signs (erythema, tissue infiltration, and keratinization) were significantly reduced. • Enhanced collagen fibre formation. • Inflammatory cytokines (TNF-α, IL-4, IL-13, and MDA) decreased substantially.	No recorded abnormality	Preclinical trial	[198]
**7.**	Omiganan loaded liposomes	2 mg	Atopic Dermatitis and Psoriasis	• Improvement in AD and psoriatic lesions. • Significant reduction in pro-inflammatory cytokines.	No recorded abnormality	Preclinical trial	[199]
**8.**	Chamomile loaded liposomes	Once daily throughout radiation therapy	Radiation dermatitis	• 76.9% patients experience dry desquamation. • Lower grades of radiation dermatitis	No recorded adverse effect	Clinical trial phase 2	[200]

### Therapeutic applications of liposomes in dermatology

#### Antifungal therapy and dermatophytosis

Liposomes have demonstrated considerable potential in enhancing the delivery and efficacy of antifungal agents for the treatment of dermatophytosis. These vesicular systems improve drug solubility, targeting, and retention in the stratum corneum, thereby augmenting therapeutic outcomes. Notably, liposome-based formulations of griseofulvin exhibit improved entrapment efficiency and controlled drug release, rendering them well-suited for sustained antifungal activity [201]. Furthermore, liposomes have been shown to interact with fungal pathogens such as Candida albicans, facilitating increased drug-liposome interaction at the infection site and enhancing antifungal efficacy [202]. Their ability to enhance drug retention in the upper layers of the epidermis further supports their utility in the management of cutaneous mycoses [202, 203].

#### Management of bacterial infections

Liposomes have also been explored for the targeted delivery of antimicrobial agents in the treatment of bacterial dermatoses, particularly those involving biofilm-associated infections [204]. Their inherent properties, including low systemic toxicity, site-specific delivery, and the capacity to penetrate biofilm matrices, enable improved local drug concentration and reduce recurrence rates [205]. For instance, liposomal polyvinylpyrrolidone (PVP)-iodine hydrogel formulations have been investigated for the treatment of impetigo contagiosa and related infective dermatoses, offering extended and controlled drug release profiles that may enhance both therapeutic efficacy and patient compliance [206].

#### Liposome-based strategies for vitiligo

Recent advancements in liposomal drug delivery have extended to the treatment of vitiligo, with several combination strategies showing promise in enhancing therapeutic response. Liposomes have been employed for the delivery of psoralen, a melanogenesis-inducing agent, and resveratrol, a potent antioxidant that mitigates oxidative stress, both of which are critical to regimentation in vitiligo [207]. Moreover, liposomal formulations have been integrated with ultraviolet (UV) light therapy, demonstrating significant depigmentation effects in clinical studies [208]. Adjunctive approaches involving topical calcineurin inhibitors or corticosteroids in conjunction with phototherapy have yielded improved regimentation outcomes [209, 210], while emerging combinations with antioxidants and laser therapies offer additional therapeutic potential, albeit with limited clinical data to date [211].

### Cosmeceutical application of liposomes

#### Anti-ageing and dermal regeneration

In cosmeceutical applications, liposomes have gained significant attention as delivery vehicles in anti-ageing formulations. These nanocarriers facilitate the dermal delivery of biomolecules and enhance their retention within the skin. Liposomal delivery systems have been shown to promote the retention of collagen within artificial membranes and elevate the expression of structural skin proteins such as collagen, keratin, and involucrin, contributing to improved skin elasticity and reduced signs of ageing [212]. Additionally, liposome-encapsulated bee venom has exhibited superior therapeutic efficacy in the management of atopic dermatitis, attributed to the downregulation of pro-inflammatory cytokines and chemokines, as well as a reduction in mast cell infiltration [212].

#### Applications in hair loss and scalp care

Liposomes are widely incorporated into hair care products, including therapeutic formulations for alopecia. Their structural resemblance to biological membranes—comprising phospholipids and cholesterol, confers enhanced biocompatibility and facilitates efficient transfollicular drug delivery [213, 214]. These properties allow for improved localization of active agents at the hair follicle site, potentially enhancing follicular health and promoting hair regrowth through sustained and targeted delivery mechanisms.

**Fig. 7:**
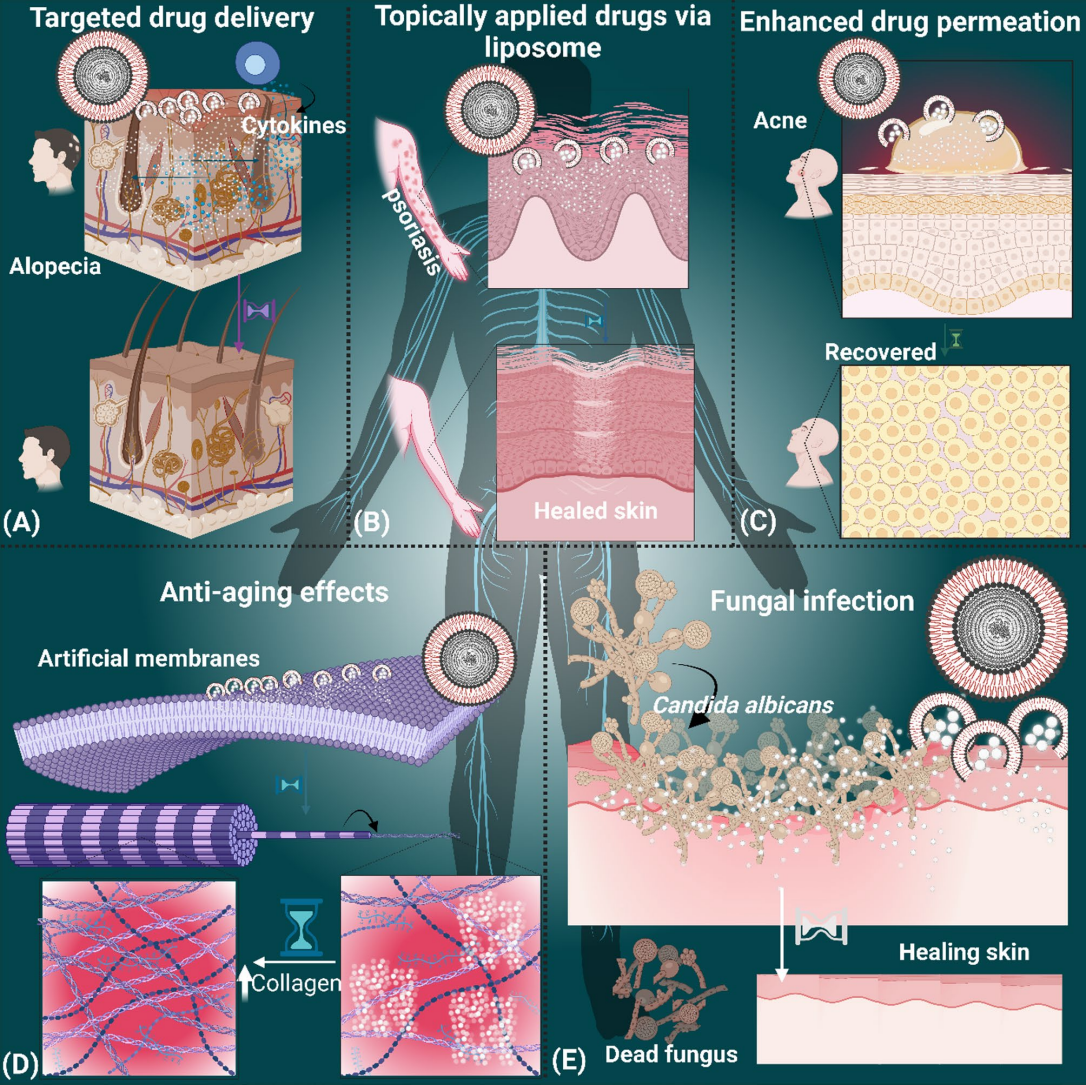
Applications of transdermal delivery of liposomes. (**A**) Targeted drug delivery of drugs encapsulated liposomes for treating alopecia. (**B**) Administering liposome-containing drugs topically (**C**) Recovery of skin from acne using liposome-based drug delivery. (**D**) Application of liposome-containing drugs on artificial membranes enhances collagen content, resulting in anti-aging effects. (**E**) Liposomes containing antifungal drugs cure for dermatophytosis

Collectively, liposome-based delivery systems represent a transformative approach in both therapeutic and cosmeceutical dermatology. By improving the stability, localization, and controlled release of active pharmaceutical ingredients, liposomes enhance the efficacy and safety profiles of topical treatments. These advancements offer promising avenues for the management of complex dermatoses and for the development of next-generation cosmeceutical products. Figure 7 illustrates the transdermal delivery mechanism of liposomes, underscoring their multifunctional role in dermatological applications.

### Role of transdermal delivery in dermatosis

Transdermal drug delivery is an alternative approach to hypodermic injection and oral delivery of drugs which offers a wide range of applications. It has numerous benefits, including better patient compliance, bypassing the first pass effect, ensuring controlled rate of drug release, and allows to swiftly halt or terminating the treatment, if required. Transdermal delivery can be used to bypass the first-pass effect, which would otherwise prematurely metabolize the drugs. This approach enhances their bioavailability, efficacy, and targeted delivery. This eliminates the need for intrusive, irritable needles, which generate medical waste, pose a danger of infection, and need to be administered by a medical expert [215]. For the proper delivery of the drug encapsulated in the liposome, it is imperative to take into consideration the properties of both the stratum corneum and the drug, while considering the site of application, body temperature, and the blood flow of the skin. There lies a narrow physiochemical window for the drug to effectively pass the stratum corneum which includes low melting point, low molecular weight of 600 Da, and water/octanol partition coefficient in the range of 1–3 [216].

The human skin, accounting for approximately 16% of total body weight, is the largest organ and serves as a primary barrier against environmental insults such as microbial invasion, UV radiation, and chemical or mechanical stress [217, 218]. Structurally, it comprises three main layers: the subcutaneous layer (hypodermis), dermis, and epidermis [219, 220]. The subcutaneous layer contains fat deposits, while the dermis consists of connective tissue. Above it lies the epidermis, an avascular layer that relies on nutrient diffusion through the dermo-epidermal junction. The outermost layer of the epidermis is the stratum corneum (SC), which consists of 15–20 layers of non-viable, flattened corneocytes embedded in a lipid matrix, and can thicken from 10 to 15 μm to 40 μm under moist conditions [221, 222]. This SC layer forms the principal barrier to drug permeation, largely due to its unique lipid composition, predominantly ceramides, cholesterol, fatty acids, and cholesteryl sulfate, arranged in bilayer structures within intercellular spaces [48, 223–225]. These structures regulate water and drug transport and can be disrupted by solvent treatments. Unlike viable epidermal layers, the SC lacks phospholipids [223]. The epidermis also features tight junction proteins that enhance barrier integrity and includes four primary cell types: keratinocytes (forming the water barrier), melanocytes (pigment production), Langerhans cells (immune defense), and Merkel cells (touch receptors) [226, 227]. Due to the formidable barrier posed by the SC, only a limited number of drugs are approved for transdermal delivery [228, 229].

Transdermal delivery allows skin penetration modifying the properties of the stratum corneum using passive or active methods. While the passive technique focuses on drug and vehicle interaction and optimization to affect the stratum corneum structure, the active approach utilizes external energy to facilitate the transportation of drugs through the skin or by physically disrupting the outermost layer of the skin, known as the stratum corneum [230, 231].

### Active method

#### Ultrasound devices

In 1950, Fellinger and Schmidt were the first to deliver the drugs utilizing ultrasound through the skin. Their findings indicate that the combination of hydrocortisone ointment and sonophoresis is effective in treating polyarthritis. Sonophoresis or phonophoresis is the application of ultrasound for drug delivery via the skin [232]. Mechanical energy is propagated longitudinally with the application of ultrasound perturbation at frequencies 20 kHz − 16 MHz with high oscillation of (compression) pressure and low (rarefaction) pressure decreasing the resistance of the skin. By modulating their frequencies, they can raise the skin’s temperature by absorbing sound waves with a frequency higher than what humans can hear [233].

Liposomes utilised with ultrasound (sonophoresis) present an effective method for improving transdermal drug administration. Low-frequency ultrasound (20–100 kHz) causes inertial cavitation, causing gas bubbles in the coupling medium to collapse near the stratum corneum, thereby generating microjets and shockwaves that disturb the lipid structure of this barrier [234]. These temporary disturbances generate hydrophilic microchannels, enhancing the penetration of liposomal vesicles and allowing the release of contained pharmaceuticals. Furthermore, steady cavitation and acoustic streaming facilitate passive diffusion and augment local fluid transfer [235]. Ultrasound application to liposome formulations can produce regulated release by cavitation-induced pore development in the lipid bilayer. In a study by Kim et al., the ultrasound-sensitive liposomes exhibited a size distribution of 81.94 nm, with a doxorubicin entrapment effectiveness of 97.1 ± 1.44%. The release of doxorubicin under ultrasound irradiation was 60% with continuous wave and 50% with optimised focused ultrasound settings [236].

#### Velocity based techniques

Velocity-based transdermal drug delivery methods include jet injection, iontophoresis, and electrophoresis, each utilising distinct physical principles to improve skin penetration. Jet injectors function by expelling high-velocity liquid jets at speeds between 100 and 200 m/s, facilitating needle-free administration of pharmaceuticals through the stratum corneum. These devices are generally propelled by compressed gas or mechanical springs and have been widely employed for the delivery of vaccinations and macromolecules, providing a minimally invasive alternative to traditional needles [187, 237]. The notion of jet injection originated in the early 1930 s, first presented by Arnold Sutermesiter.

Iontophoresis entails the utilisation of a low electric current to facilitate the transdermal delivery of charged drug-loaded liposomes, hence improving transport across dermal barriers by electrorepulsion and electroosmosis [238–240]. Electrophoresis utilises electric fields to enhance the migration of charged molecules, such as liposomal carriers, by augmenting skin permeability and facilitating active transdermal transport [241–243]. Collectively, these velocity-based methods provide accurate, non-invasive, and efficient drug administration, especially advantageous for macromolecules and formulations exhibiting low passive permeability.

#### Electroporation

it is a well-established physical technique employed for the Transdermal administration of large molecules. By subjecting cells to high-intensity electric pulses, typically ranging from 50 to 500 volts for a brief duration. This exposure results in the creation of aqueous pores within the lamellar lipid regions of the SC, thereby facilitating the drug diffusion across the skin barrier [192, 244]. This technique was discovered by Neuman et al., in 1982 [245]. This method offers immediate action, delivery of macromolecules, and insignificant or minor skin damage. It has been efficient in increasing the TDDs with varying molecular weights including small (fentanyl, orcalcein) to high molecular weight drugs weighing up to 40 kDa. Electroporation, which employs brief, high-voltage pulses, creates transitory holes in the stratum corneum, facilitating enhanced penetration of liposomes. The incorporation of anionic lipids, specifically dimyristoylphosphatidylserine (DMPS), enhances pore stability, yielding a four- to eighteen-fold increase in insulin penetration and producing more robust, linear delivery profiles [246].

#### Iontophoresis

Transdermal iontophoresis is a potential method for delivering a range of chemicals in a regulated and scheduled manner [247]. This approach has the potential to be widely employed for the administration of hydrophilic medicines. It is a non-invasive technique that enhances dermal and transdermal delivery with the application of continuous low-voltage current [248]. It is known to provide a physiologically acceptable electrical current(0.1-1.0 mA/cm^2^) using an electrode of the same polarity to drive charged permeants across the SC through its potential gradient [249, 250]. By the phenomenon of electroosmosis weakly charged molecules or uncharged molecules are transferred but for the transfer of charged molecules, electrophoresis is required. Iontophoresis utilises a mild electric current to enhance the mobility of both charged and uncharged liposomes through the skin, promoting their uptake by skin cells via endocytosis. Research indicates that liposomal formulations delivered using iontophoresis significantly enhance the transdermal transport of encapsulated proteins (such as platelet-rich plasma), propranolol, diclofenac, and peptides in both porcine and human skin, surpassing passive diffusion methods [251–253].

By integrating liposomal encapsulation with velocity-driven techniques, researchers can attain higher control over drug release, improved localisation at target areas, and increased bioavailability, rendering this strategy a potent platform for non-invasive and efficient transdermal therapies.

### Thermal approaches

#### Laser thermal ablations

hows potential for enhancing the permeability of SC, which is the outermost layer of the skin, while preserving the integrity of deeper underlying tissues. For treating skin dermatosis including pigmented lesions, laser ablation technique is widely used. The fundamental principle of laser thermal ablation is that laser irradiation causes the skin target to decompose into microscopic particles that travel away from the skin surface at a supersonic speed. As a result, laser ablation may selectively eliminate the SC without causing damage to deeper tissue. This ablation can significantly impair stratum corneum’s barrier function, allowing drugs to pass through the skin more easily [254, 255]. The structural integrity of the skin must be evaluated, especially during the high-intensity laser used for big molecular weight drugs.

Lee et al. carried out a study to enhance and regulate the penetration of peptides and associated vaccinations into the skin using erbium: yttrium–aluminium–garnet (Er: YAG) laser. Laser therapy and lysozyme antigen immunisation were performed on mouse skin in vivo. Peptide administration increased significantly when the Er: YAG laser partly ablated the SC. Despite a thin and fragmented SC laser, treated skin had 3 to 140 times more peptide than untreated skin. Laser therapy tripled blood serum antibody production without any extra chemicals or boosters [256].

Recent studies indicate that laser thermal ablation enhances the transdermal administration of liposomal formulations by promoting deeper skin penetration and enhanced drug release. Fractional CO₂ laser pre-treatment has shown improved absorption of liposome-encapsulated hydrophilic substances, including 5-carboxyfluorescein and ovalbumin, in both pig and murine skin, with a notable increase in permeability linked to the applied laser intensity [257]. The Er: YAG laser selectively ablates sections of the stratum corneum, enhancing the penetration of hydrophilic and lipophilic medicines contained in liposomes, while preserving skin viability [258]. These lasers function by generating microchannels and temporarily breaking lipid bilayers, facilitating the penetration of nanoscale carriers like liposomes into the living epidermal and dermal layers. Furthermore, the localised heat effects of laser exposure can augment the fluidity of stratum corneum lipids and facilitate regulated liposomal fusion and payload release, hence enhancing bioavailability and targeted accumulation of medicines. This method has significant potential for administering macromolecular medications and vaccinations, which are otherwise constrained by the skin’s barrier properties.

#### Radiofrequency (RF) Thermal Ablation

involves the placement of microelectrode arrays and the use of high frequency alternating current (about 100 kHz) to the specific area [256]. The narrow pathways for drug penetration in the SC are formed when the ions of the cell oscillate near the microelectrode. High frequency alternating current generates thermal energy through ionic vibration, leading to the evaporation of water and the ablution of cells, perhaps causing harm to underlying tissue. Low-cost, disposable devices can be used to transdermally distribute several hydrophilic drugs and macromolecules [259]. Microchannel’s presence is confined to the superficial layers of the skin, where the blood vessels and nerve endings are absent, resulting in a reduction of discomfort. The microchannels are rapidly filled with hydrophilic interstitial fluid within a matter of seconds. The regulation of medicine distribution is influenced by the microchannel number and depth, as well as the microelectrode parameters.

RF-induced thermal ablation has demonstrated significant potential in enhancing the transdermal delivery of liposomal drug formulations. By creating uniform and transient microchannels in the stratum corneum, RF facilitates the deeper penetration of liposomes through otherwise impermeable skin barriers. Specifically, studies have shown that RF ablation markedly increases the skin permeation of liposomal encapsulated compounds, including hydrophilic and macromolecular drugs such as insulin and interferon-α, by enabling direct access to the viable epidermis and dermis [260]. The localized heat generated by RF can also temporarily increase lipid fluidity in the stratum corneum, promoting fusion of liposomal bilayers with disrupted skin lipids and enhancing the release of encapsulated drugs [261]. Moreover, RF ablation has been used synergistically with deformable liposomes (transfersomes and ethosomes), further amplifying delivery efficiency by facilitating both mechanical passage through RF-induced microchannels and biochemical fusion with skin lipids [262]. These findings indicate that RF thermal ablation is a promising strategy for the non-invasive, site-specific delivery of liposomal drugs, especially for molecules with poor transdermal permeability under conventional methods.

### Mechanical approaches

#### Microneedle arrays

**Fig. 8:**
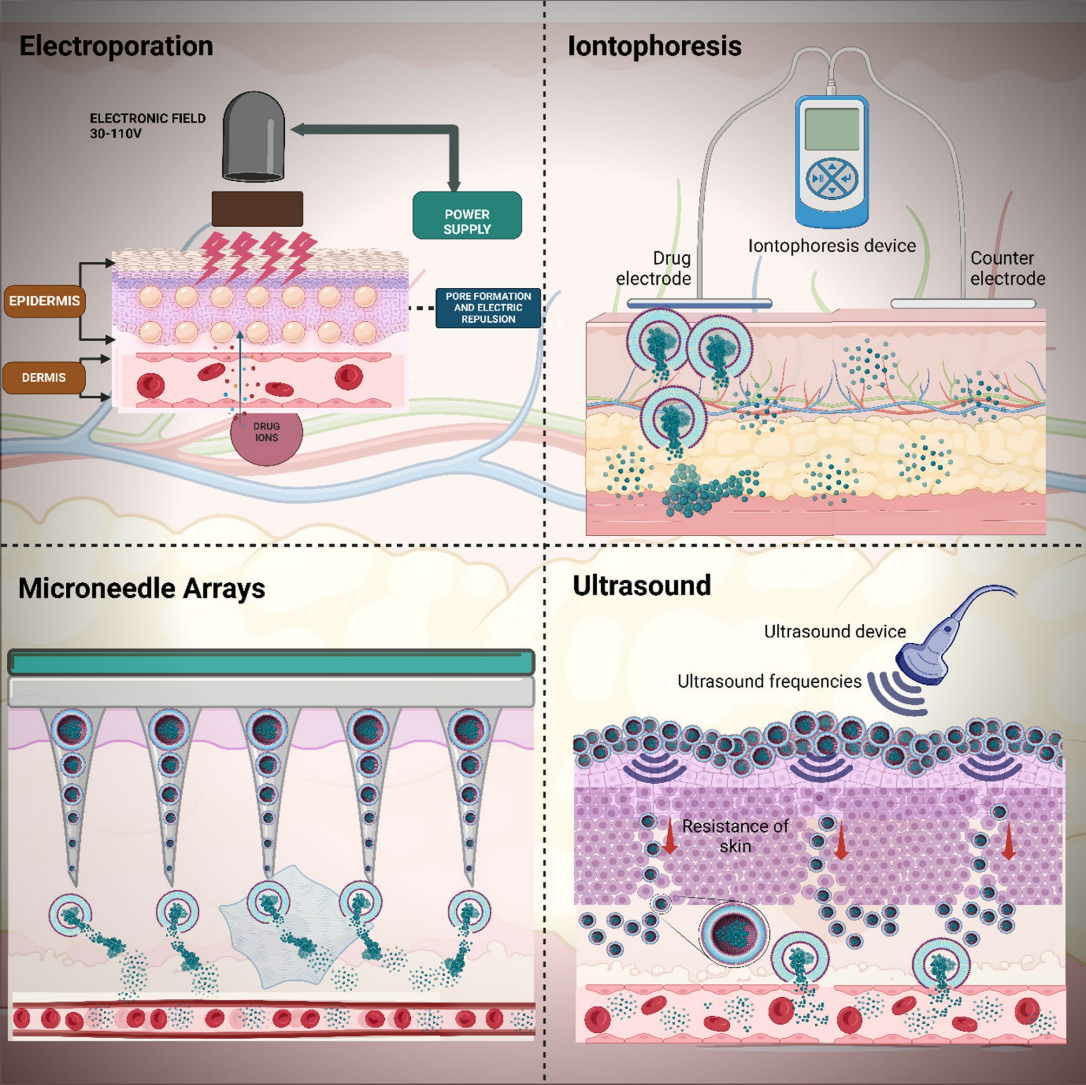
Illustration of four distinct active methods employed in transdermal drug delivery. (1) Positioned in the top left quadrant is the electroporation which involves the application of short high-voltage electrical pulses facilitating permeability for drug absorption. (2) The top right quadrant represents Iontophoresis, which employs the application of low-level electric current to facilitate moles through the skin’s lipid barrier. (3) In the bottom right quadrant is microneedle array, that provides minimally invasive micro-sized needles for the creation for transient channels in the skin. (4) Represented in the bottom right quadrant is the ultrasound devices, which employ mechanical vibrations to disrupt the SC-enhancing drug permeation through the skin

were created to alleviate the drawbacks of hypodermic needles and to administer drugs that could not diffuse through the skin using passive or externally assisted diffusion [263, 264]. MN tests were initially developed to keep the person safe from discomfort caused by hypodermic needles [265]. The Microneedle has the potential to pierce the stratum corneum, generating channels for the drug molecule to quickly diffuse beneath the epidermis. Microneedles (MN) consist of multiple projections typically arranged unilaterally on a supportive base or patch. These projections vary in base width from 50 to 250 μm, heights between 25 and 2000 μm, and diameters of tip from 1 to 25 μm [238, 266]. MN was initially conceptualized in the 1970 s by Martin S. Gerste and Virgil A. Place [267]. Figure 8 illustrates various mechanical approaches of transdermal delivery including microneedle arrays.

To produce transitory aqueous conduits MN was designed, which crossed the skin. As a result, small hydrophilic compounds to macromolecules showed an increase in the flow as well as avoided any nerve exposure when introduced into skin layers [268, 269]. MN arrays are prepared from a wide range of materials including metals (Silicon, Palladium, Titanium, etc.), natural polymers (Zein, Chitosan, Dextran, etc.), biodegradable polymers (PVP, PLGA, PLA, etc.), and non-biodegradable polymers (Alginic acid, Polyetherimide, Polyvinyl acetate, etc.) [270]. Aside from painless administration, MN arrays provide numerous advantages, including the absence of bleeding, controlled drug release, minimal pathogen introduction, reduction in transdermal dosage variability of small compounds, and concerns associated with infections [266, 271, 272].

Microneedles have shown significant effectiveness in improving the transdermal administration of liposomal medication compositions, leveraging these benefits. The microchannels created by microneedle insertion provide direct, low-resistance conduits across the stratum corneum, facilitating the penetration of intact liposomes or their contents into deeper skin layers. This is especially advantageous for hydrophilic or high molecular weight pharmaceuticals contained in liposomes, which encounter considerable permeability obstacles. Research indicates that microneedles improve the transdermal delivery and systemic uptake of liposome-encapsulated pharmaceuticals, including insulin, vaccinations, and anticancer medicines [272, 273]. Furthermore, the mechanical rupture of the skin by microneedles has been shown to facilitate the fusing of liposomal bilayers with intercellular lipids, hence enhancing the release and diffusion of the active pharmaceutical agent [274]. The integration of MNs with deformable liposomes (e.g., ethosomes, transfersomes) enhances delivery efficacy, since the pliable vesicles can navigate via microchannels and release their contents directly into the dermis [275]. Moreover, MN-mediated administration of liposomal formulations has been shown to diminish drug degradation, enhance bioavailability, and provide regulated or sustained release profiles, establishing it as a highly effective platform for both local and systemic drug delivery [276].

#### Tape stripping

is an invasive technique used for the removal of the SC by the repeated applications of adhesive tapes. It is critical to flatten the adhesive tape with the force similar to the roller while applying it to avoid the influence of recesses and creases on tape peeling [277]. The amount of SC removed depends on factors including anatomical site, pH and transepidermal water loss [278]. The pace of removal of the adhesive tape is indirectly proportional to the quantity of skin removed from the patch, which is directly proportional to the adherence of the SC to the patch [279]. While tape stripping is a simple and robust procedure, several aspects should be addressed before implementing this technique.

The mechanical extraction of stratum corneum layers via successive applications of adhesive tapes facilitates liposome penetration into deeper epidermal strata, thus enhancing the bioavailability and therapeutic effectiveness of the encapsulated pharmaceuticals. Tape stripping has been utilised as a pre-treatment to enhance medication penetration and as an analytical technique to evaluate the depth and uniformity of drug permeation into the skin. Research has shown that the absorption of liposome-encapsulated hydrophilic compounds, such as 5-aminolevulinic acid, markedly increases following tape removal, which facilitates vesicle migration into the living epidermis [280]. This approach demonstrates synergy with deformable liposomes or transfersomes, which can further navigate through the intercellular spaces of the partially removed stratum corneum, hence improving their delivery efficiency [281]. Notwithstanding its invasiveness, tape stripping is regarded as a reproducible and regulated technique for temporarily diminishing the skin barrier, rendering it a significant instrument for investigating and enhancing liposomal transdermal drug delivery systems.

### Passive method

#### Chemical enhancer

application is a widely used passive method for transdermal delivery without long-term damage to the skin. It promotes the movement of drugs into the barrier domain of the stratum corneum, leading to improved drug absorption. Enhancer has the potential to modify the properties of the formulation to promote the delivery of drugs [282]. Species interaction at the cellular level, dispersion or intercellular lipids extraction, increased fluidity of stratum corneum lipid bilayers, higher drug thermodynamic activity, and greater SC hydration are all mechanisms that support drug administration using chemical enhancers [283].

Various substances have been examined as enhancers, with regularly researched examples including alcohols (such as ethanol), fatty acids, polyalcohols, pyrrolidone, amines, amides, dimethyl sulfoxide, sulfoxides, esters, water, terpenes, surfactants, and others [284]. The chemical enhancers must be non-allergenic or non-toxic, showing compatibility with both drugs and excipients. These are usually unidirectional with rapid working activity with a predetermined duration [285]. When utilised in conjunction with liposomes, chemical enhancers such as ethanol, oleic acid, terpenes, and surfactants interact synergistically to augment the permeability of both the skin and the liposomal vesicles. Ethanol fluidizes the stratum corneum lipid bilayers and improves the deformability of liposomes, facilitating their passage through the intercellular matrix of the stratum corneum to reach viable epidermal layers [258]. Terpenes like limonene and menthol are recognised for their ability to alter the lipid organisation of the stratum corneum, generating microvoids that facilitate the passage of liposomes [286]. Surfactants such as sodium lauryl sulphate can solubilise intercellular lipids, diminishing the barrier resistance of the stratum corneum and enhancing the penetration of vesicular carriers [287]. This amalgamation of mechanisms not only improves the bioavailability of liposome-encapsulated pharmaceuticals but also facilitates the administration of larger or more hydrophilic compounds that would typically encounter difficulties in permeating the epidermal barrier. Research indicates that liposomes altered with ethanol (ethosomes) or utilised with chemical enhancers demonstrate markedly increased transdermal drug flow relative to traditional liposomes [288]. Chemical enhancers work as effective adjuncts in liposomal delivery systems, offering a controlled and efficient method to surmount the skin’s robust barrier without inflicting long-term damage.

#### Vesicular and nanocarriers

The drug-carrying vesicles in transdermal delivery including liposomes and nanoparticles are one of the most promising methods which ease skin penetration as it improves fluidizing the skin lipids, the drug portioning into the skin, and drug solubilization into the formulations [289, 290]. Ultra-flexible liposomes such as ethosomes are employed to increase penetration for poorly soluble drugs’ delivery [291]. Polymeric nanoparticles are chosen for TDDS because they easily toss chemical and physical properties, minimising drug reduction and boosting SC penetration. Self-assembling nanocarriers made of biocompatible, biodegradable, and amphiphilic copolymers, these polymeric vesicles can be natural or synthetic [292, 293].

Vesicular and nanocarrier-based approaches, especially those employing liposomes, have shown significant promise in augmenting transdermal drug delivery by boosting drug stability, penetration, and release kinetics. Liposomes, consisting of phospholipid bilayers, function as biocompatible carriers capable of encapsulating both hydrophilic and lipophilic medicines. Their flexible form facilitates merging with stratum corneum lipids, enhancing penetration into deeper skin layers [294]. Progress in vesicular formulations, including ultra-deformable liposomes (transfersomes and ethosomes), has enhanced skin permeability by allowing the vesicles to go across intercellular routes in the stratum corneum under osmotic gradients or ethanol-induced lipid fluidisation [258, 281]. Ethosomes specifically possess elevated ethanol concentrations that augment stratum corneum lipid fluidity and vesicle pliability, thus enhancing the transdermal transit of poorly water-soluble pharmaceuticals [295]. Polymeric nanoparticles, like those composed of PLGA or chitosan, provide regulated drug release and safeguard against enzymatic degradation, while improving skin penetration due to their adjustable size, surface charge, and hydrophobicity [296]. Moreover, self-assembling nanocarriers, constructed from amphiphilic copolymers, can replicate natural membrane architectures and facilitate drug delivery through diffusion or endocytosis, all while ensuring minimal cytotoxicity and elevated biocompatibility [297]. These vesicular and nanocarrier systems collectively augment the capacity of liposomes to penetrate the epidermal barrier, providing potential platforms for safe and effective transdermal medication delivery.

#### Nano-emulsion

The use of nanoemulsions to enhance drug efficacy is well established, with current research largely focused on the development of semisolid dosage forms based on nanoemulsion technology [298]. Nanoemulsions serve as effective carrier systems for delivering various substances through intact skin, which functions as a natural barrier. Their advantages stem from the nanoscale droplet size, offering high solubilization capacity, thermodynamic and kinetic stability—surpassing that of conventional dosage forms. Structurally, nanoemulsions are composed of two immiscible liquids, forming an isotropic yet heterogeneous system wherein pharmaceutical agents are encapsulated within nanodroplets [299, 300].

Sithole et al. developed nano-emulgels and nano-emulsions as an alternative to oral administration that facilitated the transdermal distribution of statins. An oil-in-water (o/w) nanostructure was developed, consisting of 2% (w/w) statin and 8% apricot kernel oil as the oil phase. The nanoemulsions were found to have a size ranging from 14.23 to 169.83 nm, whereas the size of the nano-emulgels ranged from 149.83 to 267.53 nm. Entrapment efficiency varied from 90.77 to 99.55%, confirming statin inclusion. Membrane release procedures showed that nano-emulsions released statins more than nano-emulgels, as seen by greater flux values. Ex vivo (skin diffusion) experiments indicated that nano-emulsions had relatively lower median values than nano-emulgels [301].

Incorporating liposomes into nanoemulsion systems provides the twin benefits of vesicular encapsulation and droplet-mediated increased permeability of the stratum corneum. The lipid composition of nanoemulsions disturbs the intercellular lipid structure of the skin, facilitating enhanced drug diffusion and deeper tissue penetration [302]. Moreover, nanoemulsions improve the fluidity and flexibility of liposomal membranes, hence enhancing fusion with skin layers and aiding the administration of both hydrophilic and lipophilic pharmaceuticals [303]. Moravedeh et al. conducted a study revealing that curcumin-loaded liposomal nanoemulsions exhibited superior percutaneous absorption and sustained release compared to plain liposomes or curcumin solution, hence confirming the synergistic effect of liposomes and nanoemulsions in transdermal applications [304]. The elevated kinetic stability and thermodynamic equilibrium of nanoemulsions diminish phase separation and enhance shelf life, rendering them appropriate for integration into semisolid formulations such as creams or gels for effective transdermal delivery [305]. Nanoemulsions boost the skin permeability of liposomal carriers and provide controlled and targeted distribution, hence improving therapeutic efficacy.

### Liposome as a delivery vehicle

**Fig. 9:**
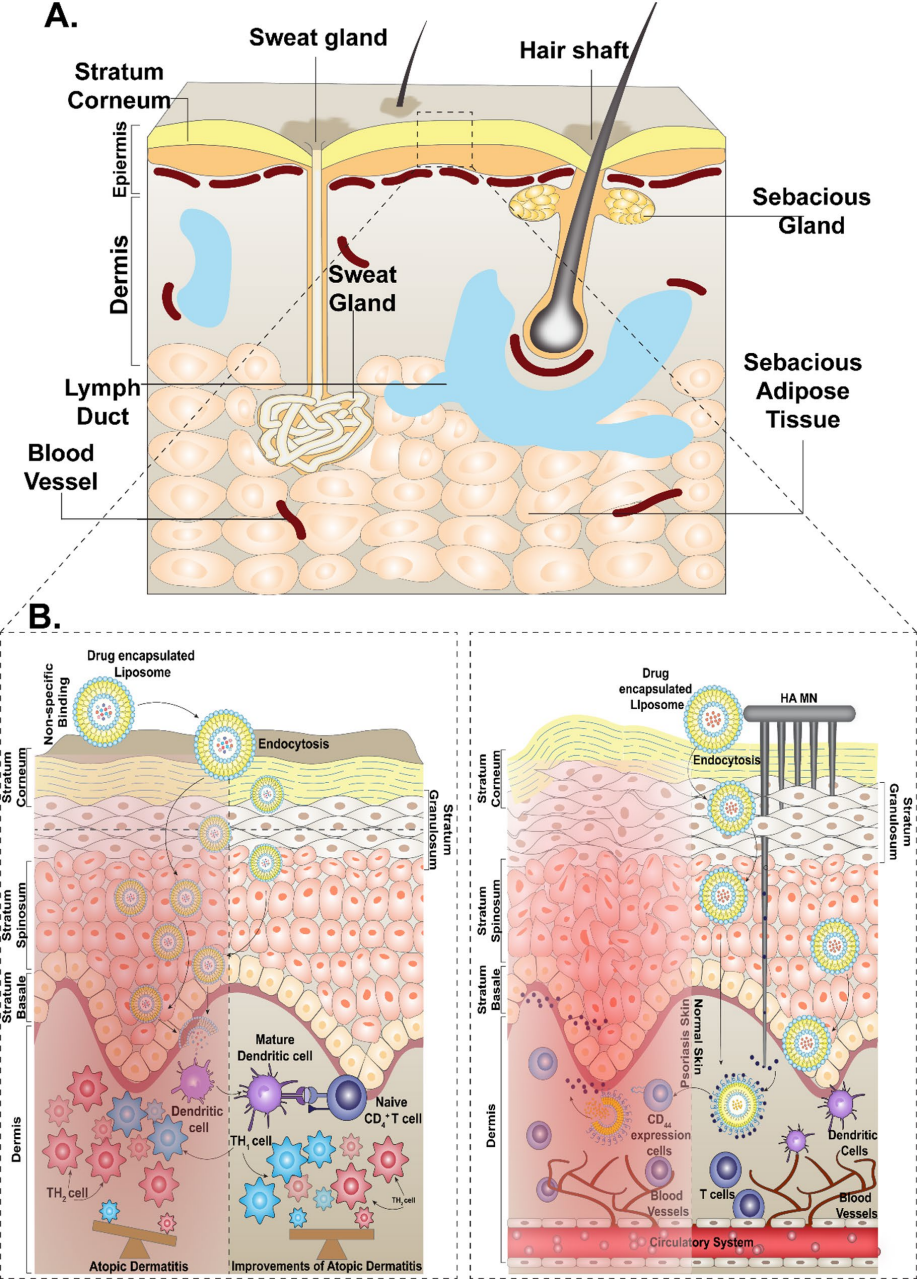
**(A)** Layers of the skin; **(B)** Possible mechanism of Liposome entry into the skin for Atopic Dermatitis treatment (Left) and Psoriasis (Right)

**Fig. 10:**
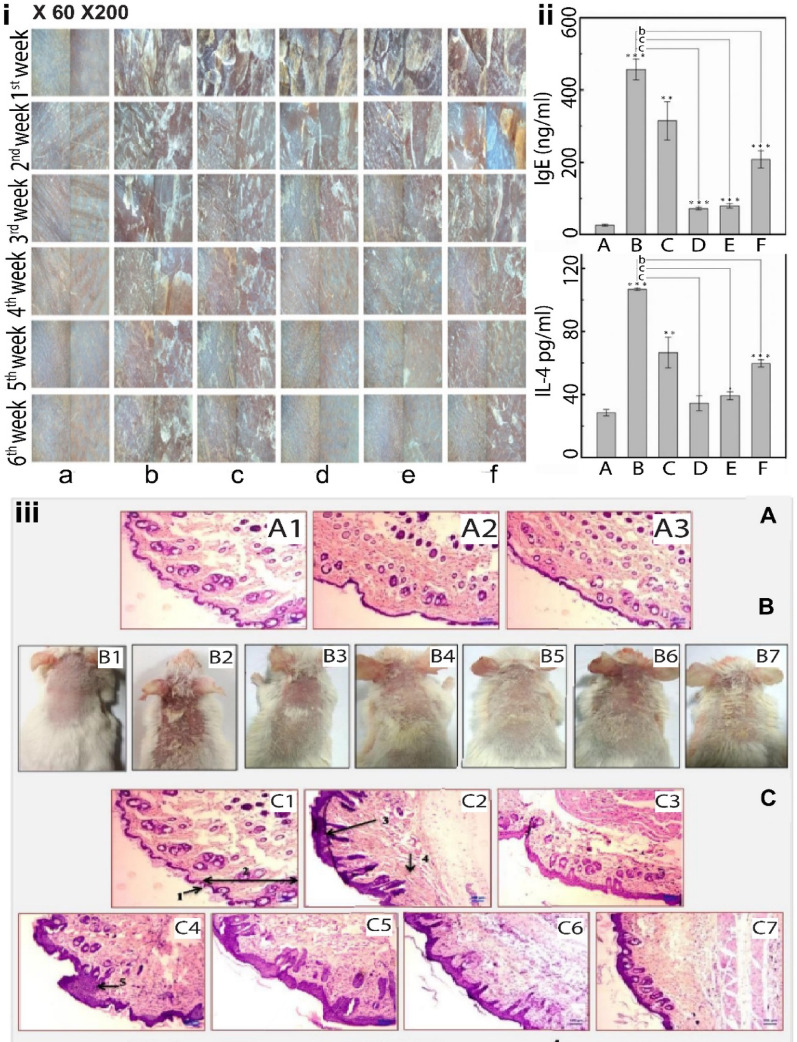
**(i)** Dorsal skin morphology of mice with DNCB-induced atopic dermatitis (AD), treated with various formulations of *H. cordata* extract (PBS, cubosomes, liposomes), compared to untreated controls and healthy skin (magnifications: 60× and 200×). **(ii)** Serum IgE and IL-4 levels in corresponding groups, indicating immunomodulatory effects of the treatments (*p* < 0.05, *p < 0.01, **p < 0.001). Adapted with permission from [309]. **(iii)** Skin compliance and histological changes in IMQ-induced psoriatic mice treated with thymoquinone (TMQ) solution and lipospheres, showing dose-dependent improvement in epidermal thickness and morphology. Adapted with permission from [310]

Liposomes due to their biphasic structure, can encapsulate both hydrophobic and hydrophilic pharmaceutical compounds. Liposomes, which are noted for their great versatility and interchangeability, are regarded as potential vehicles that can be employed in modified-release formulations, for targeting organs, or as protectors of compounds that are sensitive to pH, light, or aqueous medium [306]. Among the different types of liposomes, namely SUVs, LUVs, and MLVs, depending on the quantity and dimensions of bilayers, LUVs have the ideal build and constitution for transdermal delivery owing to their high capacity for loading drugs, efficient penetration across the SC, and inherent stability. Phospholipid bilayers in the liquid crystalline state are preferred for transdermal drug administration, and liposomal formulations depending on composition may be divided into two major groups: innovative liposomes such as flexible/deformable/elastic liposomes and standard liposomes [307]. The general mechanism of liposomal drug delivery follows the implantation of drug molecules within the aqueous core of the liposome, using the lipid bilayer, ensuring its protection from the body’s aqueous environment. The membrane deteriorates with time and the drug contents are released at the site of release [308]. Figure 9 illustrates possible mechanism of liposomal drug delivery for skin diseases such as Atopic dermatitis and psoriasis. Figure 10 shows the experimental results of drug delivery on mice for the treatment of Atopic dermatitis and psoriasis by Kwon et al. and Jain et al. [309, 310].

In 1992, Cvec et al. initially proposed the concept of ultra-deformable or flexible liposomes as an alternative to the limits posed by conventional liposomes. These are biodegradable and biocompatible containing natural phospholipids. They exhibit better encapsulation efficiencies, reaching up to 90% for lipophilic pharmaceutical compounds (PCs). Additionally, they enable the encapsulation of hydrophilic PCs with molecules of varying molecular weights [281, 311]. The ultra-deformable liposomes boost the active pharmaceutical penetration via SC using two methods. In the first process, liposomes function as vesicles that permeate the skin primarily due to their deformability and amphiphilic nature [312]. Their inherent flexibility allows them to withstand mechanical stress and facilitates their penetration into deeper layers of the skin, including the viable epidermis. Additionally, these liposomes act as permeation enhancers by interacting with and disrupting the organized lipid matrix of the SC, particularly by altering the structure of the intracellular lipid lamellae. This disruption promotes enhanced diffusion of the encapsulated drug across the skin barrier [313, 314]. In the second mechanism, liposomes function as reservoirs that release the active drug in a regulated manner at or near the skin’s surface. The drug subsequently diffuses across the stratum corneum following its concentration gradient. This technique depends on the slow release and passive diffusion of the drug molecule, perhaps augmented by the liposomal formulation and its interaction with skin lipids [32].

Pharmaceutical compounds (PCs) that are not associated with a vehicle or attached to the surface have the potential to penetrate the skin. When the PCs are attached to vesicles, the penetration occurs as the vesicle dissolute in the skin and joins the SC, resulting in the formation of structures with the lipid lamella, increasing the pharmaceutical compound’s penetration [315]. Liposomes as a delivery vehicle, adsorb to the stratum corneum’s surface and release PCs directly on the skin via adsorption or they can fuse with the SC’s lipid matrix facilitating the diffusion of PCs within the skin [316]. There’s improved sedimentation stability for stratum corneum liposomes as compared to hydroalcoholic solutions, and alcoholic-oil emulsions [317].

Phospholipid monomers can gather at electroporated sites, forming liquid microdomains within the skin, thus reversing damage induced by electroporation [318]. Also, integrating hydrophilic drugs into charged liposomes can significantly enhance their encapsulation efficiency, which is typically low.

In a study published by Rangsimawong, the effect of sonophoresis at a frequency of 20 kHz on the transdermal administration of sodium fluorescein (NaFI)using lipid-based nanocarriers including niosomes (NI), liposomes (LI), and solid lipid nanoparticles (SLN). Sonophoresis reduces the skin penetration of NaFI-loaded SLN by a factor of 6.32 and NI by a factor of 1.79, while enhancing the penetration of NaFI-loaded liposomes by a factor of 5.36. These results could be because of sonication which could lead to break the vesicle, lamella, and the solid cores of the nanocarrier. However, when the liposome lamellae degraded, the residual phospholipids diffused into the SC to actively repair the skin via adsorption while simultaneously enhancing drug penetration [319].

Once the drug has been dissolved in the vehicle of choice, diffusion of the drug occurs from the vehicle or reservoir to the skin surface. Once the drug is present on the skin surface, it can diffuse through the epidermis through two routes, either transepidermal(transcellular or intercellular) or transappendageal(sweat ducts, hair follicles, and pilosebaceous glands) [311]. For intercellular delivery, once the drug of interest penetrates the SC. It permeates the lipid domains before diffusing through both the lipid bilayers and the aqueous region [320]. In the transcellular or intracellular pathway, the permeation occurs through the corneocyte clusters within the imprecations that generate openings containing water [321]. This pathway is thus considered relatively hydrophilic, as it offers lower resistance to the permeation of hydrophilic compounds through the aqueous domains of corneocytes. The transappendageal delivery follows the penetration through a diffusional shunt provided by the hair follicles or the pilosebaceous unit [290, 322]. It is followed by the partitioning into the sebum followed by the diffusion through the lipids. From here, the drug molecule for all three delivery pathways is partitioned into the viable epidermis where it diffuses through the cellular mass of the epidermis and diffuses through the upper dermis [323]. It finally gets uptake by the capillaries or the deeper tissue entering the systemic circulation.

In research conducted by Walunj et al., the improvement of cyclosporine-loaded cationic liposomes was done to address the unfavourable physicochemical properties of Cyclosporine, a drug for immunosuppression. The positive charge because of Dioleoyl-3-trimethylammonium propane (DOTAP), on the surface of the liposome, improved its affinity for the anionic skin membrane resulting in enhanced efficiency in a psoriatic plaque model induced by imiquimod (IMQ) [324]. Yu et al., further expanded the research to enhance transdermal absorption, by incorporating cell-penetrating peptides (CPP) into the liposomes. The paracellular routes across the epithelium open because of the CPPs’ interaction with the C-terminus of the Na^+^/K^+^-ATPase beta-subunit (ATP1B1). Curcumin-CPP (Cur-CPP) liposomes had better skin penetration and cutaneous retention than Curcumin liposomes without CPPs. Cur-CPP liposomes significantly ameliorate apparent symptoms including erythema and silver scales, as well as epidermal thickness, in an IMQ-induced psoriasis mouse model [325].

#### Atopic dermatitis (AD)

is a persistent and relapsing chronic skin inflammation that causes itching, xerosis, edema, peeling, and erythema. The exact cause of this condition is unidentified however immunological dysregulation, and allergic-like reactions are thought to have a role. Elevated levels of interleukins (ILs), immunoglobulin E, and certain blood cells (such as mast cells, type 2 helper T cells, and eosinophils) are perceived in the plasma [326, 327]. Omiganan, a synthetic cationic peptide of 12 amino acids, is used to treat atopic dermatitis because it kills both gram-positive and gram-negative bacteria, and fungus. In mice with atopic dermatitis (AD) and psoriasis, gel and lotion were compared to Omiganan liposomal gel. In addition to 72% encapsulation.

efficiency, liposome formulation demonstrated 7.2% loading efficacy, 120 nm vesicle size, and − 16.2mV zeta potential. Compared to lotion and gel formulations, Omiganan liposomal gel dramatically lowered pro-inflammatory cytokine levels and enhanced AD and psoriatic lesions [199]. Kwon et al. investigated enhancing the efficacy of the water-soluble extract of Houttuynia cordata against AD by utilising lipid nanocarriers. The extract was incorporated into liposome and cubosomal solutions using a thin film hydration technique. The study revealed that cubosomes, compared to liposomes, enhanced the penetration of HCWSE into the skin and prevented further development of atopic dermatitis in hairless mice. Suspensions containing HCWSE decreased the synthesis of IgE and IL-4 while increasing the expression of IgE [309].

#### Psoriasis

is an autoimmune condition caused by genetic and environmental causes. It is characterised by silver-white scales, inflammatory cells in the epidermis, keratinocyte hyperproliferation, and lesions [328]. In an experiment conducted by Yu et al., curcumin modified with peptides (CRC-TD-Lip) was loaded into liposomes, and its effect was tested by transdermal delivery on the psoriatic area. Results showed increased uptake and inhibitory effect of CRC-TD-Lip on HaCaT cells. The modified curcumin resulted in better anti-psoriasis efficiency [325]. Thymoquinone (TMQ) has strong anti-psoriatic efficacy, but its hydrophobicity, low water solubility, and light and pH sensitivity limit its transport to the target site. Jain et al. created lipospheres with particle sizes less than 70 nm using TmQ encapsulation. Cell line studies revealed a decrease in the levels of IL-1β, IL-6, nitric oxide, TNF-α, and IL-2while in vivo research revealed better constitution and histopathological features with lower TNF-α and IL-17 in psoriatic skin and lipospheres, allowing for deeper skin penetration and target site delivery. Figure 10 represents the experimental results of drug delivery on mice for the treatment psoriasis by Jain et al. [310]. This study shows skin compliance study of the psoriatic mice skin treated with normal skin, Thymoquinone (TMQ) solution, and TMQ liposphere. TMQ lipospheres shown a greater decrease in the thickness and regularity of the epidermis, as compared to the TMQ solution. Both the solution and lipospheres treated groups showed improvement that varied depending on the dose [310].

#### Vitiligo

**Fig. 11:**
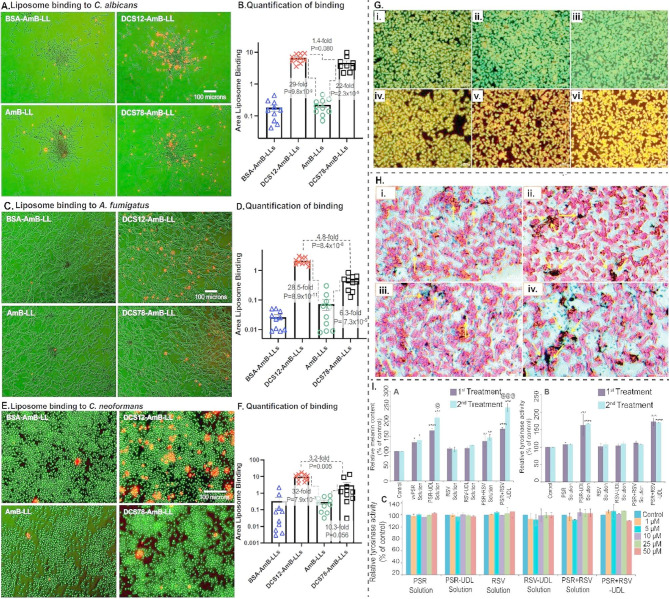
Targeted liposomal formulations (DCS12-AmB-LLs and DCS78-AmB-LLs) exhibit enhanced binding to exopolysaccharide matrices of C. albicans, **A**. fumigatus, and **C**. neoformans compared to non-targeted AmB-LLs; (**A**, **C**, **E**) exhibit fluorescent imaging while (**B**, **D**, **F**) show flow cytometry analysis. Adapted with permission from [338]. **G.** Cellular uptake of ultra-deformable liposomes (UDL) evaluated in B16F10 cells, indicating enhanced cellular internalization. Scale bar: 1000 μm. **H.** Effect of Psoralen (PSR) and Resveratrol (RSV) UDL formulations on melanin synthesis in B16F10 cells. Fontana-Masson silver staining visualized melanin as black deposits (yellow arrows) across four groups: (i) Control, (ii) PSR-UDL, (iii) RSV-UDL, and (iv) PSR + RSV-UDL. Combined PSR + RSV-UDL treatment significantly enhanced melanin deposition and tyrosinase activity, with PSR contributing predominantly. Scale bar: 1000 μm. **I.** A. Quantification of relative melanin content in treated B16F10 cells. **B**. Tyrosinase activity assay in cells across treatment groups. **C**. Cell-free tyrosinase assay assessing direct enzymatic inhibition by formulations. Results are expressed as mean ± SD (*n* = 3). Statistical significance: *p* < 0.05, *p* < 0.01, *p* < 0.001, *p* < 0.0001 vs. control. G, H, and I Adapted with permission from [208]

is a disorder characterised by developing white skin spots acquired with an unknown cause [329]. Psoralen (PSR) in conjunction with ultraviolet A (PUVA) treats vitiligo, a depigmenting skin condition, by promoting both tyrosinase activity and melanin production in melanocytes. Resveratrol (RSV) functions as a sirtuin activator and a possible antioxidant, decreasing oxidative stress that leads to inflammation in vitiligo. Resveratrol coupled with psoralen was useful in treating vitiligo. Figure 11** G**,** H**,**I** shows a study by Doppalapudi et al. on the uptake of cells to ascertain the ability of UDL to penetrate, as well as the impact of PSR and RSV formulations on melanin and tyrosinase [208].

#### Acne

is a prevalent skin issue that affects individuals globally, without being limited by ethnicity or genetics and is most frequently observed among teenagers and young adults. The blockage of keratinocytes, sebum in the sebaceous gland, and hair follicles causes this inflammatory skin illness as it restricts the sebum from reaching the skin’s surface [330]. It frequently results in developing pimples or zits on the skin’s surface, leading to redness, swelling, discomfort, and heat [331]. Madan et al., designed an approach to enable the efficient formulation of cationic liposomes for the preparation of curcumin, azithromycin, and lauric acid liposomal gels. The stability was assessed over 60 days. TNF-α and IL-1β regulate inflammatory markers responsible for acne-induced inflammation. All the liposomal formulations and the combined application of curcumin and lauric acid liposomal gels effectively reduced IL-1β release caused by P. acnes and suppressed the release of TNF-α. Therefore, when curcumin and lauric acid liposomal gels are combined for therapy, there is a notable decrease in IL-1β and TNF-α levels by around 1.9 and 2.5 times, indicating its effectiveness in treating acne [332].

Quercetin is proven to be therapeutic on cutaneous eczema, a kind of skin disease characterised by inflammation. However, quercetin has low solubility in organic solvents because of its structural features thus leading to low availability and limiting clinical applications. The preparation of quercetin-containing liposomes for gel (QU-LG) formulation proves to be efficient against eczema as it significantly reduces dermatopathological symptoms. The sodium carboxymethylcellulose (CMC-Na) present in the QU-LG can improve the flexibility of the liposome bilayer reducing the barrier function of the SC, creating channels for the drug molecule to enter for exerting its antioxidant and anti-inflammatory activities [333]. Ascorbic acid, widely used in cosmetic formulations, is delivered to the skin with negatively charged liposomes favouring better skin retention [334]. Resveratrol (3,3,3’-triydroxy-trans-stilbene), a phenol derived from the root of knotweed (Polygonium cuspidatum), found in Japan and China [335]. It is effective for treating venereal diseases purulent skin infections and mycoses. It also has wound healing and anti-cancerous activities (melanoma) [336]. To increase its bioactivity and bioavailability resveratrol is encapsulated inside liposomes which acts as an ideal solution for its activity as resveratrol is less soluble in water. Furthermore, recent findings presented by Kwon et al. suggested that co-encapsulation of resveratrol with 4-n-butyl resorcinol (4nBR) in liposomes helped in melasma treatment [337]. Additionally, Fig. 11 illustrates the study conducted by Ambati et. al., which involved the use of DCS12 and DCS78 coated liposomes on fungal exopolysaccharide matrices which are known to cause skin infections. The study focused on three different fungus species and aimed to examine the binding and quantification process. This study showed that the liposomes DCS12-Amphoterecin B-Loaded Liposome (DCS12-AmB-LLs) and DCS78-Amphoterecin B-Loaded Liposome (DCS78-AmB-LLs), targeted to DC-SIGN, exhibit a higher level of binding efficiency to the exopolysaccharide matrices of three highly dangerous fungal pathogens compared to AmB-LLs. of UDL.

## Challenges and limitations

Liposomes and lipid-based vesicles employed for encapsulating and administrating pharmaceuticals have significant potential in drug delivery, specifically in dermatological applications. The challenges associated with liposomal drug administration discuss a range of factors, including stability and the short half-life of liposomal drugs, concerns about their size and penetration, encapsulation effectiveness, skin barrier integrity, and the crucial component of patient compliance. Clinical evidence from studies shedding light on these limitations and problems reveals the complexities of optimizing liposome-based drug delivery to treat skin disorders [32].

### Limited half-life and stability

Liposomes for their limited half-life and susceptibility to degradation and fusion present significant challenges in drug delivery. Over time, liposomes can undergo degradation due to exposure to light, temperature fluctuations, and the existence of enzymes within living structures [98]. Liposomes can fuse with other cellular structures, such as cell membranes. This fusion can occur during storage, transport, or within the bloodstream, leading to the merging of their contents. Furthermore, liposomes can degrade, destabilising their lipid bilayer structure, which results in the premature release of the encapsulated drug. This sudden release can lead to suboptimal drug concentrations at the intended target site in the body, diminishing the drug’s therapeutic effect [339]. Encapsulated drugs within liposomes have been found to degrade or lose their activity before exerting their intended medicinal effects [32]. This can be especially problematic in applications where maintaining drug potency and precise delivery timing is crucial, such as cancer therapy or treating infectious diseases. Despite these challenges, liposomes remain a valuable tool in drug delivery, and ongoing research seeks to maximize their potential while minimizing their burdens [340].

### Size and penetration

Liposomes come in various sizes, and selecting the proper size is crucial for efficient skin penetration. The size of liposomes and their impact on skin penetration and stability present complex challenges that must be addressed for optimal drug release and penetration. Smaller liposomes, typically in the nanometre range, tend to penetrate the skin more effectively due to their ability to navigate its various barriers. However, there’s a trade-off with stability – smaller liposomes are more susceptible to aggregation, fusion, and cargo leakage, which may reduce their efficacy [341]. On the other hand, giant liposomes are generally more stable but may not penetrate the skin as efficiently. Researchers and formulators face the challenge of tailoring liposome size to suit specific dermatological applications, considering the skin’s anatomy and the therapeutic agent’s properties [341]. Smaller liposomes are preferred for deep penetration, while more giant liposomes might be more appropriate for superficial treatments. Additionally, finding ways to enhance the stability of smaller liposomes, perhaps through surface modifications or the inclusion of stabilizing agents, is an ongoing area of research. However, liposome size is not the only factor affecting skin penetration and efficacy. The lipid composition, charge, and surface properties of liposomes also play a significant role. Therefore, it is essential to consider these factors in conjunction with size to develop liposomal formulations that maximize drug delivery and therapeutic outcomes in dermatology [32].

### Encapsulation efficiency

Encapsulation efficiency is another critical factor in liposome-based drug delivery systems, and its limitations pose challenges in pharmaceutical development. Liposomes encapsulate drugs in lipid bilayers or aqueous cores, depending on their characteristics. However, drug release kinetics and therapeutic results vary because not all drugs can be encapsulated successfully. One of the primary factors affecting encapsulation efficiency is the nature of the drug itself. Liposomes are tailored to encapsulate hydrophobic or hydrophilic drugs, but the structural compatibility between the drug and the liposome is crucial [342]. Drugs that do not readily integrate into the liposomal structure may result in lower encapsulation rates, leading to unpredictable drug release kinetics. Clinical studies have highlighted these variations in encapsulation efficiency, underscoring the need for careful formulation and optimization in liposome-based drug delivery. In cases where encapsulation efficiency is low, patients may not receive the intended dose of the drug, leading to suboptimal treatment efficacy [343].

Conversely, high encapsulation efficiency can result in drug overdosing, potentially leading to adverse side effects. Techniques such as modifying liposome composition, optimizing drug-loading methods, and designing drug-specific liposomes are being explored to enhance encapsulation [344]. Ongoing research efforts are focused on enhancing encapsulation efficiency’s reliability and consistency to ensure better patient therapeutic outcomes.

### Skin barrier integrity

Skin barrier integrity plays a crucial role in the effectiveness of liposome-based drug delivery systems, and challenges associated with compromised skin barriers in patients with dermatological diseases can significantly impact successful drug delivery. One primary challenge when dealing with compromised skin barriers is the decreased ability of the skin to function as a protective barrier, which is seen in patients with dermatological diseases such as psoriasis, eczema, or burns [345]. Disruptions in the stratum corneum often characterize this compromised barrier, the outermost layer of the epidermis responsible for maintaining skin integrity. These disruptions can be due to inflammation, excessive dryness, or other pathological processes associated with skin diseases. Liposome-based drug delivery systems rely on the intactness of the skin barrier to effectively transport drugs to the target site within the skin or to allow systemic absorption. When the skin barrier is compromised, liposomes may not adhere to the skin surface as efficiently, and drug release from liposomes may not occur as expected [346]. This can lead to a decrease in the effectiveness of drugs and an unexpected treatment outcome.

### Regulatory hurdles

Liposomes, although recognised for their promise to improve transdermal drug delivery, encounter numerous hurdles and restrictions that impede their wider clinical and commercial use. A notable obstacle is regulatory challenges, since the intricacy of liposomal formulations creates ambiguities in characterisation, quality assurance, and long-term stability, so rendering regulatory approval processes more rigorous. Regulatory bodies like the FDA and EMA necessitate thorough physicochemical characterisation, stability information, and proof of therapeutic equivalency, which can be challenging for these heterogeneous systems [124].

### Reproducibility and scalability of liposomes

A significant restriction pertains to the reproducibility and scalability of liposome synthesis. The shift from laboratory-scale to industrial-scale production frequently results in variability in vesicle size, lamellarity, encapsulation efficiency, and batch consistency, largely due to the sensitivity of liposome characteristics to process parameters like hydration rate, extrusion pressure, and lipid composition. Advanced methods such as microfluidization and high-pressure homogenisation enhance outcomes but necessitate intricate equipment and stringent quality assurance standards, hence elevating manufacturing complexity [2].

### Cost-effectiveness

Cost-effectiveness continues to be a key issue, as the raw materials (such as high-purity phospholipids and cholesterol), sterile manufacturing facilities, and specialised machinery considerably increase production costs relative to traditional formulations. Moreover, the necessity for cold chain storage and restricted shelf life exacerbate logistical challenges, imposing limitations in resource-constrained environments and affecting market feasibility [347].

### Patient adherence

Ultimately, patient adherence may be influenced by the physical properties of the formulation. Certain liposomal transdermal systems may possess oily textures or necessitate certain application methods, potentially diminishing user convenience. Furthermore, if regular application or occlusion is necessary to sustain therapeutic levels, patient adherence may diminish, especially in chronic treatment contexts [348].

## Future perspective

The future of transdermal drug delivery of liposomes for skin dermatosis holds immense promise and can revolutionise the course of medication and management of various dermatological conditions. To fully utilise the potential of liposomes in treating skin dermatosis, it is imperative to focus on several key aspects. Dermatosis encompasses many disorders, including eczema, psoriasis, acne, and various inflammatory skin diseases.

First and foremost, research and development efforts must be directed towards tailoring liposomes for specific skin conditions to maximise therapeutic outcomes by customising their lipid composition, size, charge, and surface modification. Advanced liposome vesicles, known for their elasticity, flexibility, and deformability, carry various therapeutic agents. The ability to release therapeutic compounds variably from these carriers makes them promise for future delivery systems [349]. Further investigation should prioritise the advancement of liposomal delivery vehicles, emphasising overcoming their limitations and directing efforts towards achieving targeted and precise actions. Additionally, innovation in liposomal technology should extend to incorporating cutting-edge active ingredients. Developing scalable and cost-effective manufacturing techniques is essential to make liposome-based dermatological products accessible to a wider population. This can be achieved through advancements in microfluidics, lyophilisation, and automation, allowing for the mass production of liposomes with consistent quality.

Developing user-friendly formulations that are easy to apply, non-greasy, and aesthetically pleasing can significantly enhance patient adherence to drug treatment regimens [350]. Ensuring liposomal products do not cause discomfort or inconvenience will improve patient outcomes and overall satisfaction. To address skin dermatosis, future liposomal transdermal delivery should incorporate artificial intelligence and data-driven methodologies. AI can assess extensive datasets including skin conditions, treatment response, and genetic characteristics to identify optimal liposomal compositions [351]. Modified liposomes, such as transfersomes, niosomes, ethosomes, and invasomes, have been developed to further enhance drug delivery across the skin [352]. However, further research and clinical trials are needed to completely explore the potential of liposomes in the treatment of dermatophytosis and other dermatological conditions [353].

Traditional treatments for dermatosis often rely on corticosteroids, which can have detrimental side effects [354]. Liposomes provide an opportunity to deliver alternative compounds, such as natural antioxidants, antimicrobial peptides, and anti-inflammatory agents. It can also encapsulate gene therapy vectors, opening the potential for personalized treatments. Integrating novel and safe therapeutic agents within liposomes is crucial for future exploration, offering patients more effective and side-effect-free treatment options. With the increased use of hyaluronic acid (HA) in the pharmaceutical industry, there can be new ways to explore the potential methods to integrate HA into nanoparticles and liposomal transdermal systems. This can be beneficial for treating anti-inflammatory illness, as HA improves both permeability and biocompatibility [355]. Ruxolitinib, the first topical JAK inhibitor cream has been approved by the FDA for treating mild to moderate apoptotic dermatosis [356].

One of the biggest variations in the different results in publications is because of the varied physiochemical features of liposomes including size, the charge, the composition and the drugs used for the studies. The different additives added to the liposomes for efficient drug delivery including ethanol, penetration enhancers and edge activators also modify the characteristics of the lipid bilayer [357]. Eventually, regulatory bodies and healthcare stakeholders must work together to guarantee the safety and effectiveness of liposomal formulations, while also establishing a structure for their authorization and extensive utilisation. These investigations should include assessments of skin permeability, drug release kinetics, and compatibility [358]. Long-term testing is necessary to establish the effectiveness of liposome-based therapies in comparison to conventional therapy. The provision of regulatory assistance promotes innovation within the liposomal area and supports the successful implementation of liposomal research in pragmatic clinical settings.

## Conclusion

Transdermal drug delivery through liposomal formulation has seen many advancements with the emergence of nanotechnology. Moreover, the synergy between liposomal technology and emerging techniques such as immunotherapy and gene therapy holds great potential for recolonizing dermatological treatment strategies. Liposomes provide a flexible method for delivering drugs by enclosing hydrophilic and lipophilic molecules. This approach reduces toxicity, improves drug distribution throughout the body, and prolonged therapeutic benefits. The advancement of flexible and deformable liposomes broadens the range of drug distribution, accommodating various physiological situations with enhanced tolerance. In the future, it is necessary to do further study to improve nanotechnology-based methods in clinical dermatology. Liposomes are a potential tool that may be used to enhance the effectiveness and safety of dermatological treatments.

## Data Availability

No datasets were generated or analysed during the current study.
